# Endothelial cell-derived SDF-1α elicits stemness traits of glioblastoma *via* dual-regulation of GLI1

**DOI:** 10.7150/thno.108843

**Published:** 2025-09-08

**Authors:** Ye Yuan, Xudong Liu, Liwen Kuang, Shixue Yang, Lihong Wang, Jiao Wang, Sen Wei, Zexuan Yan, Qinghua Ma, Juan Lei, Yu Zhou, Yu Chen, Jiongming Chen, Tao Luo, Kaidi Yang, Mengsi Zhang, Yongsheng Li

**Affiliations:** 1 Department of Medical Oncology, Chongqing University Cancer Hospital, Chongqing 400030, China.; 2 School of Medicine, Chongqing University, Chongqing 400044, China.; 3 Institute of Pathology and Southwest Cancer Center, Southwest Hospital, Third Military Medical University (Army Medical University), Chongqing 400038, China.; 4 Department of Oncology, Hainan Hospital of Chinese People's Liberation Army General Hospital, Sanya, Hainan Province 572013, China.; 5 Bioengineering College of Chongqing University, Chongqing 400030, China.

**Keywords:** glioma stem cell, SDF-1α, GLI1, perivascular niche, cell-cell interaction, stemness regulation.

## Abstract

**Background:** Glioma stem cells (GSCs) play a critical role in the poor treatment outcomes observed in glioblastoma (GBM) patients. A primary focus of current glioma research is understanding the maintenance of stemness in GSCs and their interactions with the tumor microenvironment. In GBMs, the perivascular niche serves as a protective environment for GSCs, contributing to tumor recurrence. However, the molecular mechanisms that sustain this reservoir remain poorly understood.

**Methods:** The analysis of single-cell transcriptional data in GBM was conducted to identify signaling pathways in endothelial cells (ECs) that promote stemness traits in glioma cells. Histological staining and the IvyGAP dataset were utilized to evaluate the anatomical microenvironment of glioma. The molecular mechanisms underlying the maintenance of stemness in GSCs, influenced by ECs, were assessed using ELISA, Western blotting, quantitative reverse transcription polymerase chain reaction (qRT-PCR), in vivo ubiquitination assays, and other molecular biology experiments. An orthotopic xenograft model was employed to examine the stemness phenotype of GBM cells in the presence of ECs, as well as the synergistic effects of GSK690693 and AMD3100 in inhibiting GBM cells.

**Results:** We found that GSCs are located in close proximity to microvessels, and we identified the CXCL12-CXCR4 signaling pathway in ECs as a promoter of stemness traits in glioma cells. GBM cells can transition to a stem-like state in response to stromal cell-derived factor-1α (SDF-1α) secreted by ECs. This transition activates the CXCR4-mediated AKT/NF-κB signaling pathway, leading to the subsequent upregulation of glioma-associated oncogene homolog 1 (GLI1), a key transcription factor for maintaining stemness. Furthermore, we discovered that SDF-1α influences the turnover of GLI1 protein in GBM cells by modulating GLI1-associated polyubiquitin chains through the phosphorylation of the deubiquitinase USP28 at serine 67. This modification enhances the stemness-maintaining properties of GLI1 via both transcriptional regulation and protein quality control mechanisms. Preclinical studies indicated that the combination of the CXCR4 antagonist AMD3100 and the AKT inhibitor GSK690693 synergistically inhibits GBM cell progression.

**Conclusions:** Our findings unveil a novel signaling axis between ECs and tumor cells that directly impacts the acquisition of stemness traits, suggesting that targeting this pathway could represent a promising therapeutic strategy against GBM.

## Introduction

Malignant gliomas are prevalent tumors of the central nervous system (CNS) that significantly impact human health. While a combination of surgery, radiotherapy, and chemotherapy has been utilized to enhance the survival of glioblastoma (GBM) patients in recent years, the prognosis for most patients remains suboptimal. Challenges such as postoperative recurrence, radiotherapy insensitivity, and chemotherapeutic resistance persist 1,2. Recent research indicates that these issues are closely linked to glioma stem cells (GSCs) 3. GSCs, a small subset of tumor cells within gliomas, possess a strong capacity for self-renewal, tumor initiation, and potential for multi-lineage differentiation 4. Numerous studies have suggested that the aggressive growth, resistance to chemo-radiotherapy, and high recurrence rates in gliomas are associated with the biological characteristics of GSCs 5. The microenvironment, or niche, of GSCs is a complex and hostile environment comprising supporting stromal cells, secreted factors, extracellular matrix, blood vessels, vascular-controlling signaling pathways, and immune cells, all of which promote the maintenance of stemness traits in neighboring GSCs 6,7 Understanding the interactions between GSCs and their niche represents a crucial frontier in current glioma research.

GBM is believed to originate from neural stem cells (NSCs) that acquire mutations and migrate from the subventricular zone (SVZ) in the adult human brain 8. Increasing evidence suggests that GSCs may derive from NSCs, as both share similar capabilities for cell renewal and multipotency 9. However, it remains unclear whether GSCs can also arise from alternative sources, given the continuous flow of GSCs into differentiated populations in order to maintain a steady reservoir of GSCs. Initial investigations into this hypothesis indicate that non-progenitor cells can transform into GSCs due to genetic and epigenetic changes, or through dedifferentiation processes that confer phenotypic plasticity to the tumor cell population 10-12. The exact mechanisms underlying dedifferentiation are still not fully understood. GSCs are frequently located in perivascular regions, creating a distinctive microenvironment of cancer stem cells (CSCs) 13. The emergence of CSCs is significantly influenced by the extracellular niche, which presents metabolic challenges 14. We propose that the vascular component in gliomas can induce neighboring non-GSCs to acquire stem-like properties through specific regulatory mechanisms. Given that gliomas exhibit extensive microvascular proliferation and that GSCs are often found in close proximity to endothelial cells (ECs), there are mutual interactions between GSCs and ECs that regulate GSC stemness, heterogeneity, invasion, and resistance to therapy. Understanding the signaling pathways involved in the vascular regulation of GSCs is crucial not only for comprehending GSC development and maintenance but also for developing innovative therapies targeting GSCs.

ECs in various tumors, including glioma, squamous cell carcinoma of the skin, and colorectal cancer, have been demonstrated to facilitate the proloferation of CSCs. This facilitation can occur through direct interactions with CSCs or by secreting paracrine signals such as nitric oxide and transforming growth factor-β (TGF-β), which activate important signaling pathways, including Notch and AKT 15,16. Inhibition of tumor angiogenesis has been shown to effectively suppress the growth of GSCs; however, the precise mechanisms underlying this effect require further investigation. Recent studies indicate that ECs secrete chemokines that engage specific receptors on CSCs. This engagement promotes site-specific invasion, metastatic processes, and resistance to chemoradiotherapy in GSCs, highlighting the critical role of ECs in maintaining the stemness of GSCs 17.

The understanding of cellular heterogeneity and tissue architecture in glioma has primarily have derived from histology, bulk sequencing, low-dimensional hypothesis-driven studies, and experimental model systems 18. Single-cell RNA sequencing (scRNA-Seq) offers promising opportunities to comprehensively characterize the cellular composition of tumors, offering new insights into cell biology, disease origins, and drug responses 19. In this study, we aimed to investigate the cellular architecture and intercellular communication in GBM by constructing a high-resolution single-cell transcriptional atlas. Our objective was to elucidate the molecular mechanisms that underpin the maintenance of stemness in GSCs as influenced by ECs, while also exploring their potential clinical therapeutic significance. Additionally, we sought to understand the interactions between ECs and neighboring non-GSCs, which may mitigate the acquisition of critical stemness properties, thereby opening promising avenues for GBM treatment.

## Results

### A high-resolution single-cell transcriptional atlas identifies CXCL12-CXCR4 signaling pathway in EC-GSC communication

**Figure 1:**
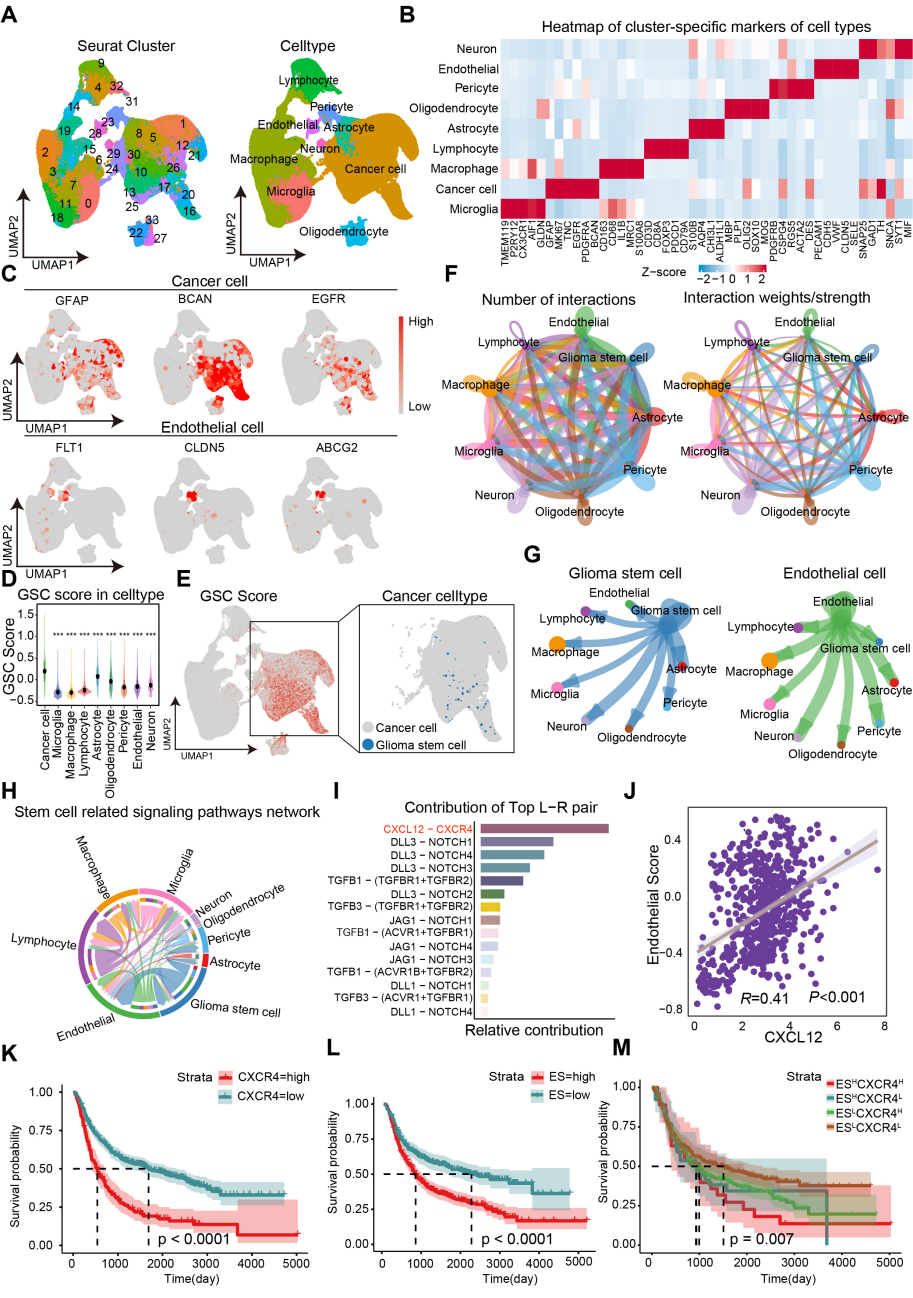
** Identification of CXCL12-CXCR4 signaling pathway in EC-GSC communication. (A)** Visualization of cell clusters after cell clustering and marker gene identification based on UMAP algorithm. **(B)** Heatmap of cluster-specific markers of cell types. **(C)** Visualization of typical markers in cancer cells and ECs. **(D)** The GSC score in each cell type. **(E)** Visualization of GSC score and GSCs sperate in cancer cell. **(F)** The number and total interaction strength of interactions between cells in glioma. **(G)** The interaction strength of ECs and CSCs with other celltypes. **(H)** The interaction of stemness-related signaling pathways between cells in gliomas. **(I)** Receptor-ligand analysis between ECs and tumor stem cells. **(J-M)** Correlation analysis of endothelial cell scores with CXCL12. Prognostic survival analysis of CXCR4, endothelial cell score with CXCR4 and endothelial cell score in glioma patients.

The cellular architecture of GBM and the maintenance mechanisms underlying the maintentance of stemness were investigated through the analysis of dataset GSE182109. A total of 232,076 single cells that passed quality control (Figure S1) were annotated using canonical lineage markers, with further validation through established gene signatures. Utilizing graph-based uniform manifold approximation and projection (UMAP), we identified 34 high-confidence cell clusters. Based on the expression of canonical gene markers, glioma clinical classification and nine major cell types were distinguished: astrocytes (10,819, 4.66%), cancer cells (77,081, 33.21%), endothelial cells (2,121, 0.91%), lymphocytes (22,222, 9.58%), macrophages (71,625, 30.86%), microglia (36,745, 15.83%), neurons (1,605, 0.69%), oligodendrocytes (6,144, 2.65%), and pericytes (3,714, 1.60%) (Figure 1A). Putative cell type identities were determined by examining differentially expressed genes in each cluster (Figure 1B). Cells exhibiting high expression of BCAN, GFAP, and EGFR were categorized as cancer cells, while endothelial cells displayed elevated levels of FLT1, CLDN5, and ABCG2 (Figure 1C). Given that the GSC state is not a stable clonal subpopulation but rather a plastic state induced by the tumor microenvironment, we scored cells based on classic GSC markers (PROM1, SOX2, NES, OLIG2, BMI1, NANOG, POU5F1) and defined the top 2% of cancer cells with the highest stemness scores as GSCs (Figure 1D-E). To investigate cell-cell communications in GBM, we utilized CellChat to infer interactions between different cell types (Figure 1F). The analysis revealed a greater number and strength of communications between GSCs/ECs and other cell types, indicating a dynamic and complex GSC microenvironment (Figure 1G).

To further investigate the potential mechanisms underlying the maintenance of stemness in GSCs, we examined the network of stem-related signaling pathways (CXCL, BMP, TGFβ, NOTCH, WNT) network involved in cell-cell communication. Among the top candidates identified in our screening were the CXCL12-CXCR4 ligand-receptor interactions (Figure 1G-I). We analyzed 18 endothelial-related genes to derive an Endothelial Score, which included SPARC, CD34, EMCN, A2M, CDH5, ABCG2, TM4SF1, CLEC14A, FLT1, ITIH5, ADGRL4, APOLD1, PECAM1, ITM2A, NOSTRIN, VWF, ABCB1, and CLDN5. Interestingly, we observed a significant positive correlation between CXCL12 (SDF-1α) and the Endothelial Score in GBM, suggesting that ECs may play a role in supporting the maintenance of stemness traits in GSCs (Figure 1J). Furthermore, elevated expression levels of CXCR4 and the Endothelial Score were associated with a poorer prognosis in GBM (Figure 1K-L). By establishing cutoff values for the Endothelial Score and CXCR4 expression, we categorized glioma patients into four distinct groups. Notably, the group with high Endothelial Score and high CXCR4 expression showed the worst prognosis compared to the other three groups, indicating that the Endothelial Score-CXCR4 axis could serve as a prognostic marker in gliomas (Figure 1M). Our findings highlight the critical role of ECs in regulating stemness traits in glioma.

### ECs promote stemness phenotype of GBM cells

**Figure 2:**
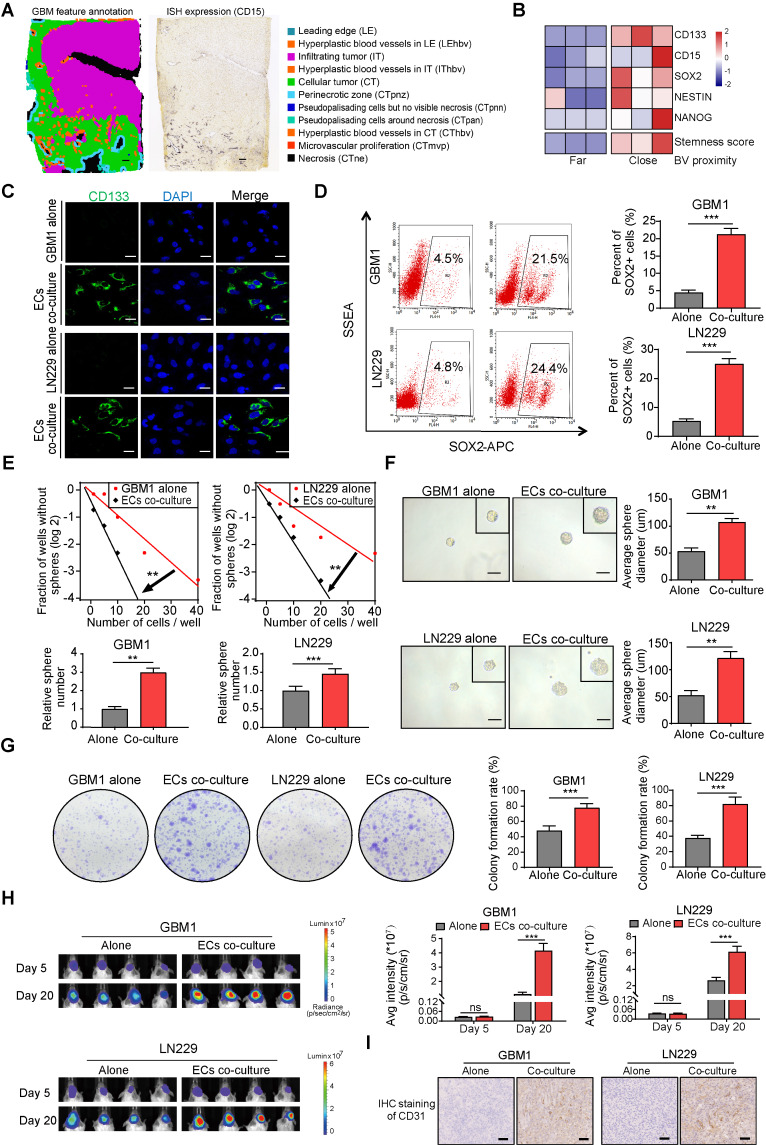
** ECs promote stemness phenotype of non-GSCs *in vitro* and *in vivo*. (A)** Distribution pattern of stem cell marker CD15 in human glioma samples. Left panel depicts tumor feature annotation for the human glioblastoma sample in the Ivy Glioblastoma Atlas Project. Right panel shows the in situ Hybridization (ISH) result in which black signals indicate the expression level of CD15. Scale bar = 500 μm. **(B)** The heatmap illustrating expression levels of stem cell markers and stemness score grouped by blood vessel proximity of glioma cells (E-MTAB-6882). BV (blood vessel). **(C)** Representative immunofluorescence images showing CD133 expression in GBM cells co-cultured with ECs compared to the control group, with nuclei stained using DAPI. **(D)** The proportion of SOX2^+^ glioma cells in both the co-culture with ECs and the control group was assessed using flow cytometry. **(E)** Limiting dilution sphere-forming assays were conducted on GBM cells either alone or co-cultured with ECs. **(F)** Representative images of tumor spheres and quantification of sphere diameters in GBM cells, both alone and co-cultured with ECs, are presented. Scale bar = 100 µm. **(G)** Representative images and quantification of colony formation rates in GBM cells, either alone or co-cultured with ECs, are shown. **(H)** *In vivo* bioluminescent imaging and quantification of xenograft tumor burdens in mice bearing either EC co-cultured or control GBM cells are illustrated. **(I)** Representative images of IHC staining of CD31 in xenografts from mice bearing either EC co-cultured or control GBM cells are provided. Scale bar = 40 um. Data are means ± SD. ns: no significance; **, *P* < 0.01; ***, *P* < 0.001.

To investigate the spatial distribution of GSCs in human GBM samples, ISH data from the IvyGAP data portal was collected. Analysis of CD15 expression, a commonly used marker for GSCs, revealed that GSCs are predominantly located near hyperplastic blood vessels (Figure 2A). Immunofluorescence staining further showed that CD133 positive cells formed a ring with higher density within a 100 μm radius of microvessels compared to the outer areas, confirming the perivascular localization of GSCs (Figure S2A-B). By categorizing the perivascular region into two distinct zones based on their proximity to tumor cells—far and close—it was found that stemness markers (CD15, CD133, SOX2, NES, and NANOG) exhibited higher expression levels in regions closer to blood vessels. This spatial pattern aligns with the ssGSEA enrichment score for the GSC gene collection and suggests a potential stemness-promoting role of ECs (Figure 2B). Furthermore, co-culturing CD133^-^ glioma cells, which are identified as non-GSCs, with ECs led to a significant increase in CD133 expression, as demonstrated by fluorescence staining (Figure 2C). Flow cytometry further confirmed that ECs induced a stemness phenotype in non-GSCs, evidenced by an elevated population of SOX2^+^ cells (Figure 2D). Classic transcription factors associated with GBM stemness, such as SOX2, OCT4, and NANOG, were significantly upregulated after co-culture with ECs, in line with the increased CD133 levels (Figure S3A). To investigate the impact of ECs on the stemness phenotypes of non-GSCs, we conducted a limiting dilution sphere-forming assay using GBM cells alone or co-cultured with ECs. Our findings demonstrate a significant enhancement in self-renewal potential and tumor sphere diameter when GBM cells were co-cultured with ECs (Figure 2E-F). Furthermore, clone formation assays revealed a significant increase in either the diameter of colonies or the colony formation rate within the EC co-culture group (Figure 2G). To more accurately assess the stemness phenotype of GBM cells in the presence of ECs, we developed an orthotopic xenograft model by implanting tumor cells either alone or in conjunction with ECs at a 1:1 ratio, consistent with the IHC staining of CD31 from the xenografts (Figure 2I). At the conclusion of the study, we observed enhanced tumor growth and upregulation of CD133 and SOX2 in xenografts derived from co-cultured GBM cells (Figure 2H and Figure S3B). Additionally, mice with co-cultured GBM cells exhibited significantly shorter survival times compared to the control group (Figure S3C).

### EC-secreted SDF-1α enhances stemness of non-GSCs through CXCR4

**Figure 3:**
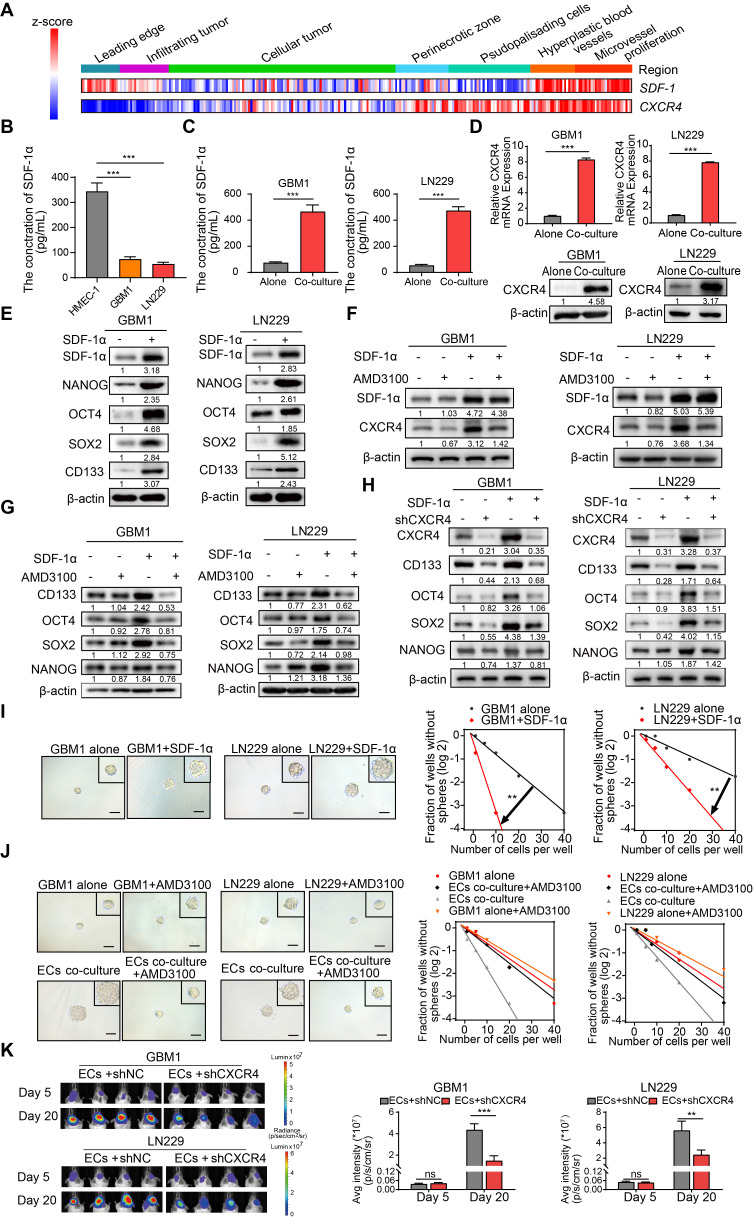
** ECs secreted SDF-1α enhances stemness of non-GSCs through CXCR4. (A)** A heatmap depicts the expression levels of SDF-1 and CXCR4 across IvyGAP GBM samples, categorized by histological type, with gene expression standardized to the same scale. **(B-C)** ELISA assays of SDF-1α levels in the supernatants of ECs, GBM cells, and GBM cells co-cultured with ECs. **(D)** qRT-PCR (upper panel) and Western blot assays (lower panel) were employed to assess CXCR4 expression in both ECs co-cultured with GBM cells and control GBM cells. **(E)** Western blot evaluating the expression levels of SDF-1α, SOX2, OCT4, NANOG, and CD133 in GBM cells treated with SDF-1α (100 ng/mL). **(F)** Western blot assay of SDF-1α, CXCR4 in GBM cells treated with SDF-1α (100 ng/mL), AMD3100 (10 μM), or both. **(G)** Western blot assay of SOX2, OCT4, NANOG, and CD133 in GBM cells under the same treatment conditions. **(H)** CXCR4, SOX2, OCT4, NANOG, and CD133 expression in GBM cells treated with SDF-1α (100 ng/mL)/shCXCR4, either alone or in combination, was evaluated *via* Western blot. **(I)** Limiting dilution sphere-forming assays were conducted on GBM cells alone and those treated with SDF-1α (100 ng/mL), with representative images of the resulting tumor spheres provided. **(J)** Limiting dilution sphere-forming assays were also performed on ECs co-cultured with control GBM cells, both with and without AMD3100 treatment, along with representative images of tumor spheres. **(K)** *In vivo* bioluminescent imaging and quantification of xenograft tumor burdens were conducted in mice bearing ECs co-cultured with shCXCR4/shNC GBM cells. Data are means ± SD. ns: no significance; **, *P* < 0.01; ***, *P* < 0.001.

SDF-1α, a chemokine recognized for its ability to enhance the migration of stem and progenitor cells, including endothelial progenitor cells (EPCs) 20,21, is constitutively expressed in ECs and plays a pivotal role in carcinogenesis and angiogenesis 22,23. Co-expression of CXCR4 and SDF-1α was identified in specific tissue regions associated with Hyperplastic blood vessels (HBVs) and microvascular proliferation (Figure 3A). Furthermore, the supernatant from cultured ECs exhibited elevated levels of secreted SDF-1α compared to GBM cells, and the presence of ECs augmented the concentration of SDF-1α in the supernatant of cultured GBM cells (Figure 3B-C). The SDF-1α secreted by ECs led to increased expression of its receptor, CXCR4, as well as stemness markers like NANOG, OCT4, SOX2, and CD133 (Figure 3D-E). To investigate the impact of the SDF-1α/CXCR4 axis on the stemness traits of GBM, we introduced AMD3100 (plerixafor), a CXCR4 antagonist, to SDF-1α-treated GBM cells. A significant increase in CXCR4 expression was observed following treatment with SDF-1α. However, this effect was diminished by the co-administration of AMD3100, suggesting that SDF-1α not only functionally activates CXCR4 but also upregulates its protein expression, potentially establishing a positive-feedback regulatory loop within the SDF-1α/CXCR4 axis (Figure 3F). Moreover, we noted that this treatment resulted in a decreased expression of stemness markers (Figure 3G). Furthermore, we knocked down CXCR4 in SDF-1α-treated GBM cells and observed a significant reversal of stemness-related genes, tumor sphere diameter, and self-renewal potential (Figure 3H and Figure S4A-B). Functional assays demonstrated that SDF-1α enhanced the self-renewal capacity of GBM cells, resulting in the formation of larger and more numerous tumor spheres (Figure 3I). However, this stemness-promoting effect was diminished by AMD3100 in the presence of co-cultured endothelial cells with GBM cells (Figure 3J). We also established an orthotopic xenograft model by implanting ECs alongside CXCR4-knockdown or control tumor cells at a 1:1 ratio. We observed diminished tumor growth in xenografts derived from co-cultured CXCR4-knockdown GBM cells (Figure 3K). These results highlight the significant role of the SDF-1α-CXCR4 axis in enhancing the stemness of perivascular non-GSCs.

### ECs endow non-GSCs with stemness owing to GLI1 transcriptional regulation by SDF-1α/CXCR4 axis

**Figure 4:**
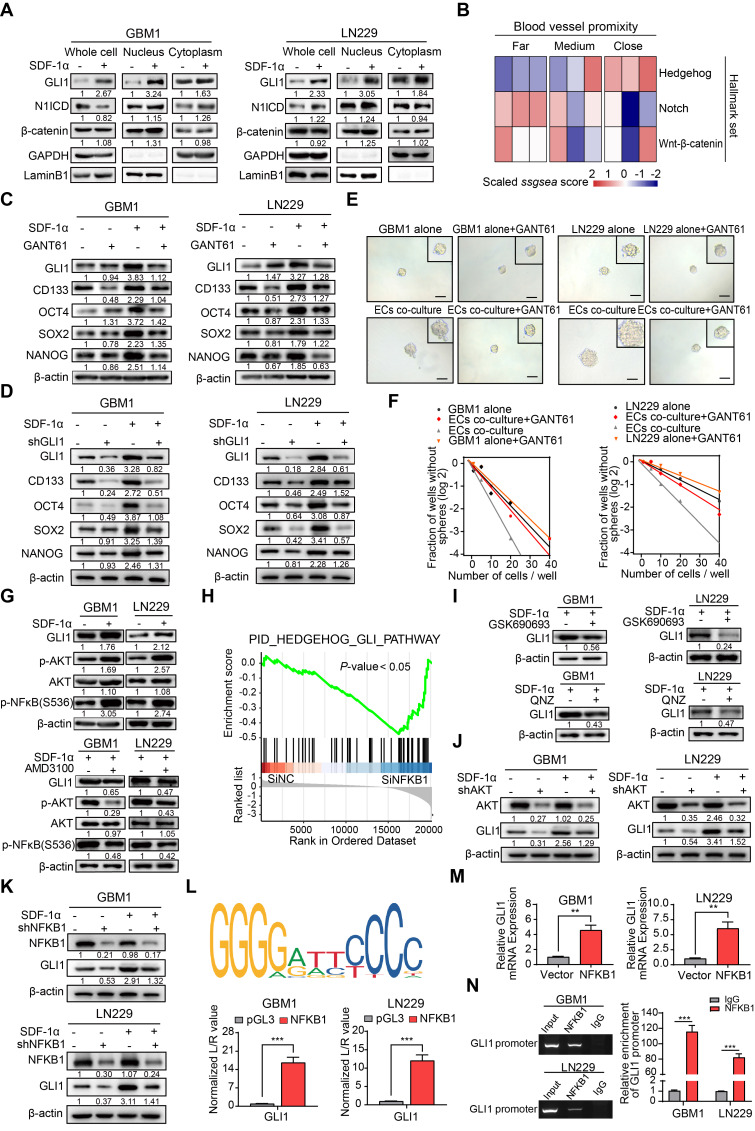
** ECs endows non-GSCs with stemness owing to transcriptional activation of GLI1 by AKT/NF-κB signaling pathway. (A)** Western blot assays were conducted to evaluate the expression levels of GLI1, N1ICD, and β-catenin in GBM cells, both in isolation and following treatment with SDF-1α, utilizing nuclear-cytoplasmic extraction methods. **(B)** A heatmap depicting the single-sample gene set enrichment analysis (ssGSEA) scores of stemness-related gene sets was generated, categorized by the proximity of glioma cells to blood vessels (E-MTAB-6882). **(C)** Western blot assays assessed the expression of GLI1, CD133, OCT4, SOX2, and NANOG in GBM cells treated with either SDF-1α (100 ng/mL) /GANT61 (5 μM) alone or in combination. **(D)** The expression of GLI1, CD133, OCT4, SOX2, and NANOG was evaluated in GBM cells treated with SDF-1α (100 ng/mL) /shGLI1, both independently and together. **(E-F)** Limiting dilution sphere-forming assays were performed on ECs co-cultured with control GBM cells, treated with GANT61 (5 μM) or not, alongside representative images of tumor spheres. **(G)** Western blot assays tested the expression levels of GLI1, p-AKT, AKT, and NFKB1 in GBM cells, both alone and following treatment with SDF-1α (100 ng/mL), with or without AMD3100 (10 μM). **(H)** GSEA plots illustrated the enrichment results of the Hedgehog-GLI gene set in control GBM cells compared to GBM cells with NFKB1 knockdown. **(I)** Western blot assays evaluated the expression of GLI1 in SDF-1α-treated GBM cells, with or without GSK690693 (2 μM)/QNZ (100 nM). **(J)** The expression levels of GLI1, CD133, OCT4, SOX2, and NANOG were assessed in GBM cells treated with SDF-1α (100 ng/mL)/shAKT, both independently and in combination. **(K)** Western blot assays evaluated the expression of GLI1, CD133, OCT4, SOX2, and NANOG in GBM cells treated with SDF-1α (100 ng/mL)/shNFKB1, both alone and in combination. **(L)** The binding site of NFKB1 on the promoter region of GLI1 was identified through analysis of the JASPAR database, and luciferase reporter assays were conducted to test the transcriptional activation of GLI1 by NFKB1. **(M)** qRT-PCR assays assessed the expression of GLI1 in NFKB1-transfected GBM cells compared to control cells. **(N)** ChIP assays confirmed that NFKB1 directly binds to the promoter region of GLI1. Data are means ± SD. ns: no significance; **, *P* < 0.01; ***, *P* < 0.001.

Given the role of the SDF-1α/CXCR4 axis in maintaining the stemness signature in neural stem/progenitor cells, we conducted nuclear-cytoplasmic extraction to investigate the downstream targets of SDF-1α/CXCR4 signaling in GBM. Our study focused on three highly conserved signal transduction pathways involved in stemness regulation: Notch, Hedgehog, and Wnt-β-catenin. We observed a significant upregulation of GLI1, a key transcriptional effector in the Hedgehog signaling pathway, in GBM cells treated with SDF-1α (Figure 4A). Interestingly, single-cell sample gene set enrichment analysis (ssGSEA) revealed a high enrichment score for the Hedgehog signaling pathway near blood vessels, indicating its association with angiogenic function (Figure 4B). *In vitro* experiments showed that the addition of the GLI1/2 transcription factor inhibitor, GANT61, to SDF-1α treated GBM cells led to reduced expression of stemness markers (Figure 4C). Furthermore, the stemness-promoting effect was diminished by the disruption of GLI1 in SDF-1α-treated GBM cells (Figure 4D). Functional assays demonstrated a decrease in self-renewal capacity in endothelial cells co-cultured with GBM cells when treated with GANT61 and GLI1 disruption (Figure 4E-F and Figure S5A-B). In addition, β-catenin exhibited mild upregulation in the nucleus of SDF-1α-treated GBM cells. We aimed to investigate whether the Wnt-β-catenin pathway also promotes the SDF-1α-dependent stemness phenotype. In subsequent validation experiments, we observed that XAV-939, a β-catenin antagonist, had minimal inhibitory effects on the SDF-1α-induced stemness phenotypes in GBM cells (Figure S6A-D).

To further elucidate the molecular mechanism, our study investigated how the SDF-1α/CXCR4 complex influenced the upregulation of GLI1. Activation of AKT/NF-κB signaling was observed upon SDF-1α stimulation, consistent with previous report 24, and this effect was attenuated by the CXCR4 antagonist AMD3100 (Figure 4G). Analysis of multiple GBM datasets revealed a positive correlation between NFKB1 and GLI1 at the mRNA expression level (Figure S7). This finding is further supported by GSEA, which demonstrated reduced enrichment of Hedgehog-GLI1 signaling following NFKB1 knockdown in GBM cells (Figure 4H). To further investigate the role of AKT/NF-κB signaling in regulating GLI1, we utilized GSK690693, a PI3K/AKT inhibitor, and QNZ, a selective NF-κB inhibitor, while silencing AKT/NFKB1 in SDF-1α pretreated GBM cells. The results confirmed a decrease in GLI1 expression (Figure 4I-K). Using JASPAR databases, we identified high-confidence predicted binding sites of NFKB1 in the promoter region of GLI1. Subsequent luciferase reporter and qRT-PCR assays validated that overexpression of NFKB1 (p50) significantly impacted GLI1 at the transcriptional level (Figure 4L-M). Additionally, CHIP assays revealed that NFKB1 exhibited significant binding to the promoter region of GLI1 (Figure 4N). These findings suggest that the proximity to endothelial cells confers stemness properties to GSCs through GLI1-mediated transcriptional regulation by AKT/NF-κB signaling pathway.

### Post-translational modifications of GLI1 protein by SDF-1α in GBM Cells

**Figure 5:**
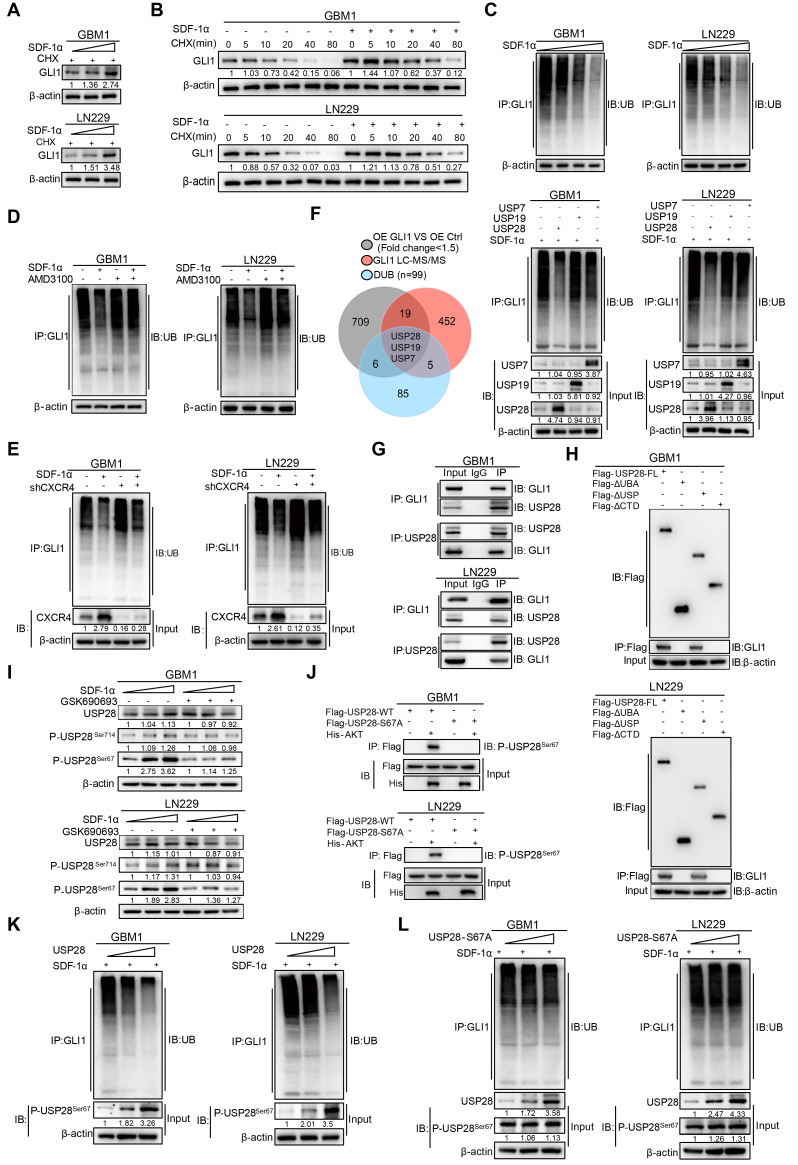
** Regulation of GLI1 protein turnover by SDF-1α in GBM cells. (A)** Western blot assay tested the expression of GLI1 in GBM cells treated with different concentrations of SDF-1α recombinant protein and CHX. **(B)** CHX pulse-chase assay tested the expression of GLI1 in GBM cells treated with SDF-1α recombinant protein or not at the indicated time.** (C)*** In vivo* ubiquitination assays of polyubiquitin chains of GLI1 in GBM cells treated with different concentrations of SDF-1α recombinant protein. **(D)*** In vivo* ubiquitination assays tested the polyubiquitin chains of GLI1 in GBM cells treated with SDF-1α recombinant protein and AMD3100 (10 μM) alone or together.** (E)** *In vivo* ubiquitination assays were conducted to evaluate the polyubiquitin chains of GLI1 in GBM cells treated with SDF-1α recombinant protein, either alone or in combination with shCXCR4. **(F)** Conjoint analysis of RNA-seq data obtained from GLI1 overexprssion and control GBM cells, GLI1 MS data and DUB geneset to identify USP7, USP19 and USP28. *In vivo* ubiquitination assays tested polyubiquitin chains of GLI1 in SDF-1α treated GBM cells which forced expression of USP7, USP19 and USP28 respectively. **(G)** Co-immunoprecipitation analyses were performed to investigate the interaction between endogenous GLI1 and USP28 in GBM cells.** (H)** Co-immunoprecipitation analyses were utilized to identify the GLI1 interacting domain within the USP28 protein.** (I)** Western blot assay tested the expression of USP28, P-USP28^Ser714^, P-USP28^Ser67^ in GBM cells treated with varying concentrations of SDF-1α recombinant protein, with and without the addition of GSK690693 (2 μM). **(J)** *In vitro* kinase assay demonstrated that AKT directly phosphorylated wild-type USP28 at serine 67 (P-USP28^S67^). **(K)*** In vivo* ubiquitination assays tested polyubiquitin chains of GLI1 in GBM cells with gradient overexpression of USP28. **(L)** *In vivo* ubiquitination assays tested polyubiquitin chains of GLI1 in GBM cells with gradient overexpression of USP28-S67A. Data are means ± SD. *, *P* < 0.05; **, *P* < 0.01; ***, *P* < 0.001.

To further address the molecular mechanisms by which SDF-1α enhances GLI1 expression, we introduced SDF-1α recombinant protein in a gradient to GBM cells. Our findings revealed a significant increase in GLI1 mRNA expression, consistent with the previously established role of the SDF-1α-AKT/NF-κB signaling axis in facilitating the transcriptional activation of GLI1 (Figure S8A). Notably, the application of cycloheximide (CHX), a protein synthesis inhibitor, resulted in the accumulation of GLI1 protein levels in SDF-1α-treated GBM cells, thereby highlighting an additional layer of complexity regarding the post-translational modification of GLI1 in GBM (Figure 5A). Moreover, CHX pulse-chase assays confirmed a significantly prolonged half-life of GLI1 following treatment with SDF-1α recombinant protein (Figure 5B and Figure S8B). *In vivo* ubiquitination assays revealed a significant reduction in GLI1-linked polyubiquitin chains following treatment with SDF-1α recombinant protein (Figure 5C). This effect could be partially reversed by AMD3100 treatment, which disrupts CXCR4 (Figure 5D-E). To elucidate the mechanisms underlying the polyubiquitination modifications of GLI1, we conducted a conjoint analysis of RNA-seq data from GLI1 overexpression and control GBM cells, along with GLI1-IP mass spectrometry data and a DUB gene set, which led us to identify USP7, USP19, and USP28 as potential regulators of GLI1 deubiquitination. In subsequent validation experiments, we observed that only the overexpression of USP28 significantly decreased GLI1-linked polyubiquitin chains in GBM cells (Figure 5F). Furthermore, a physical interaction between endogenous USP28 and GLI1 was confirmed through co-immunoprecipitation (Figure 5G). Additionally, we found that GLI1 bound to both full-length USP28 and a USP-domain truncated version of USP28, suggesting that USP28 deubiquitinates GLI1 protein in GBM cells (Figure 5H and Figure S8C).

We subsequently investigated the role of SDF-1α in promoting the deubiquitination of GLI1 by USP28 in GBM. Emerging literature suggests that the phosphorylation of USP28 at serine 67 and serine 714 by various kinases enhances its enzymatic activity 25. Given that previous results indicated SDF-1α could activate the AKT/NF-κB signaling pathway, we hypothesized that this signaling axis might phosphorylate USP28. Notably, an increase in USP28 phosphorylation at serine 67 was observed following SDF-1α stimulation, an effect that was diminished by the PI3K/AKT inhibitor GSK690693 (Figure 5I). Furthermore, we observed that AKT directly phosphorylated wild-type USP28 at serine 67 (P-USP28^S67^), whereas the S67A-mutated USP28 (USP28-S67A) did not undergo phosphorylation, as demonstrated in an *in vitro* kinase assay (Figure 5J). *In vivo* ubiquitination assays revealed a significant reduction in GLI1-linked polyubiquitin chains in GBM cells overexpressing USP28, whereas such chains were scarcely detectable in USP28 S67A point mutants (Figure 5K-L and Figure S8D). These findings highlight the role of SDF-1α in modulating GLI1 protein turnover and underscore the complexity of post-translational modifications (PTMs) involved in the Hedgehog-GLI1 signaling pathway.

### Synergistic effects of GSK690693 and AMD3100 on inhibiting migration and proliferation of GBM cells

**Figure 6:**
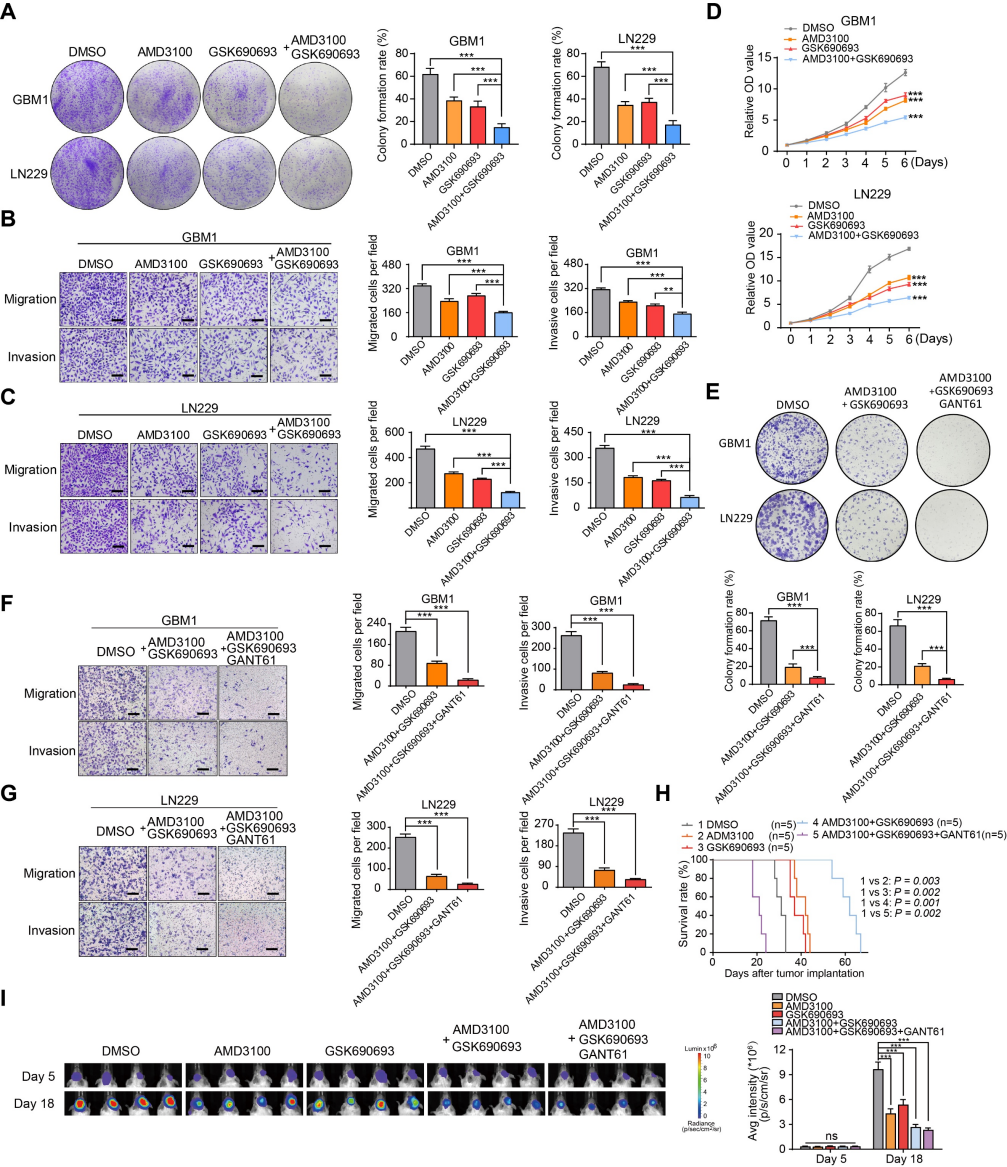
** Synergistic effect of AMD3100 and GSK690693 impairs the GBM progression. (A)** Representative images and quantification of clone formation levels in GBM cells treated with GSK690693 and AMD3100 alone or together.** (B-C)** Representative images and quantification of transwell migration and invasion assays in GBM cells treated with GSK690693 and AMD3100 alone or together. **(D)** Growth curves of GBM cells treated with GSK690693 and AMD3100 alone or together tested by CCK-8 assays.** (E)** Representative images and quantification of clonogenic formation rates in GBM cells treated with either a dual-drug combination of GSK690693 and AMD3100 or a triple-drug combination of GSK690693, AMD3100, and GANT61 are presented. **(F-G)** Representative images and quantification of transwell migration and invasion assays in GBM cells treated with dual-drug combination (GSK690693 and AMD3100) or triple-drug combination (GSK690693, AMD3100 and GANT61). **(H)** Kaplan-Meier survival analysis of the mice bearing xenograft tumors of tumor/endothelial cells mixture (at a 1:1 ratio), which treated with GSK690693, AMD3100 and GANT61 alone or together. **(I)*** In vivo* bioluminescent images and quantification of xenograft tumor burdens in mice bearing xenograft tumors of tumor/endothelial cells mixture (at a 1:1 ratio), which treated with GSK690693, AMD3100 and GANT61 alone or together. Data are means ± SD. ns: no significance; *, *P* < 0.05; **, *P* < 0.01; ***, *P* < 0.001.

To validate the therapeutic significance, we evaluated the cytotoxic effects of GSK690693 and AMD3100 on GBM cells both *in vitro* and *in vivo*. Through transwell chamber migration, invasion, and clone formation assays, we observed a synergistic effect of the combination treatment in inhibiting colony formation, migration, and invasion of GBM cells compared to individual drug treatments (Figure 6A-C). The CCK-8 assay confirmed a significant suppression of the proliferative capacity of GBM cells with combination therapy (Figure 6D). Previous results indicated that GANT61 significantly reduced the self-renewal capacity of endothelial cells co-cultured with GBM cells. Based on these findings, we hypothesized that a combination treatment involving GSK690693, AMD3100, and GANT61 would produce a more potent therapeutic effect. In our in vitro validation experiments, the triple-drug combination (GSK690693, AMD3100, and GANT61) exhibited superior tumor suppression efficacy compared to the dual-drug combination (GSK690693 and AMD3100) (Figure 6E-G). However, in an orthotopic xenograft model utilizing a 1:1 mixture of tumor cells and endothelial cells, treatment with GSK690693, AMD3100, GANT61, or their combinations resulted in a significant reduction in tumor burden and an extension of overall survival in the dual-drug combination group, rather than in the triple-drug combination group. This finding suggests potential biological toxicity risks associated with triple-drug combinations (Figure 6H-I). Collectively, these findings suggest that the combination of GSK690693 and AMD3100 effectively attenuates the progression of GBM.

## Discussion

Glioblastoma is an incurable disease characterized by a compartment of GSCs that are closely associated with proliferative vascular components. The recurrence of glioblastoma is partly due to the therapeutic resistance exhibited by GSCs. Despite the development of innovative treatments targeting GSCs, such as rindopepimut 26, ibrutinib 27, and chimeric antigen receptor (CAR) T-cell therapy 28, the curative outcomes have generally been unsatisfactory. This may be attributed to the dynamic nature of GSCs within the TME, which involves processes like differentiation (*e.g*. astrocytic and oligodendrocytic conversion) and trans-differentiation (*e.g.*, the emergence of neural, stromal, or vascular-like cells), as well as the generation of new GSCs from non-GSCs. The 'seed and soil' theory suggests that the interaction between cancer cells (the seed) and the surrounding environment (the soil) is a driving force in oncogenesis 29. Recent research has emphasized the significance of TME niches, such as perivascular, hypoxic, and fibrotic niches, in supporting the formation and maintenance of CSC subpopulations. In the GBMs, the perivascular niche acts as a protective environment for GSCs, thereby contributing to tumor recurrence. However, the molecular mechanisms that sustain this reservoir remain poorly understood. Our study reveals that ECs promote a stemness phenotype in non-GSCs through the SDF-1α-CXCR4-GLI1 regulatory axis. This finding underscores the critical role of SDF-1α released by ECs in maintaining the GSC reservoir, potentially explaining the observed enrichment of GSCs in the perivascular region.

Current evidence strongly suggests that GSCs may originate from NSCs located in the SVZ. Driver mutations in GBM gradually accumulate in these NSCs, leading to their malignant transformation. NSCs in the SVZ interact closely with blood vessels, receiving signals from ECs or pericytes that enhance their proliferative and tumor-initiating capabilities. While a small proportion of GSCs may arise from the migration of NSCs from the SVZ, the majority of GSCs (approximately 60% of the tumor compartment) are found within the glioma mass 30. This suggests that another possible source of GSCs is the dedifferentiation of non-GSCs, a crucial mechanism in maintaining the GSC reservoir. While GSC migration may contribute to perivascular niche occupancy, our functional assays, both *in vitro* and *in vivo*, demonstrate that ECs facilitate the acquisition of stemness in non-GSCs.

The resistance of GBMs to chemo-radiotherapy and its high rates of relapse indicate the presence of a persistent population of tumor cells exhibiting stem-cell properties. Studies have shown that stemness in GBM is not confined to the original GSCs but can also be acquired by differentiated tumor cells in response to environmental changes or therapeutic stress 14. For example, radiation has been shown to induce cancer cell dedifferentiation into a stem-like phenotype both *in vitro* and in tumor-bearing mice, thereby contributing to radiotherapy resistance in GBM. Additionally, characteristics of stemness can be enhanced under conditions of hypoxia or nutritional stress. Notably, similar dedifferentiation processes have been observed in oligodendrocyte-like cells 14 and astrocyte cells 31, suggesting that this property is not exclusive to a specific tumor lineage.

**Figure 7:**
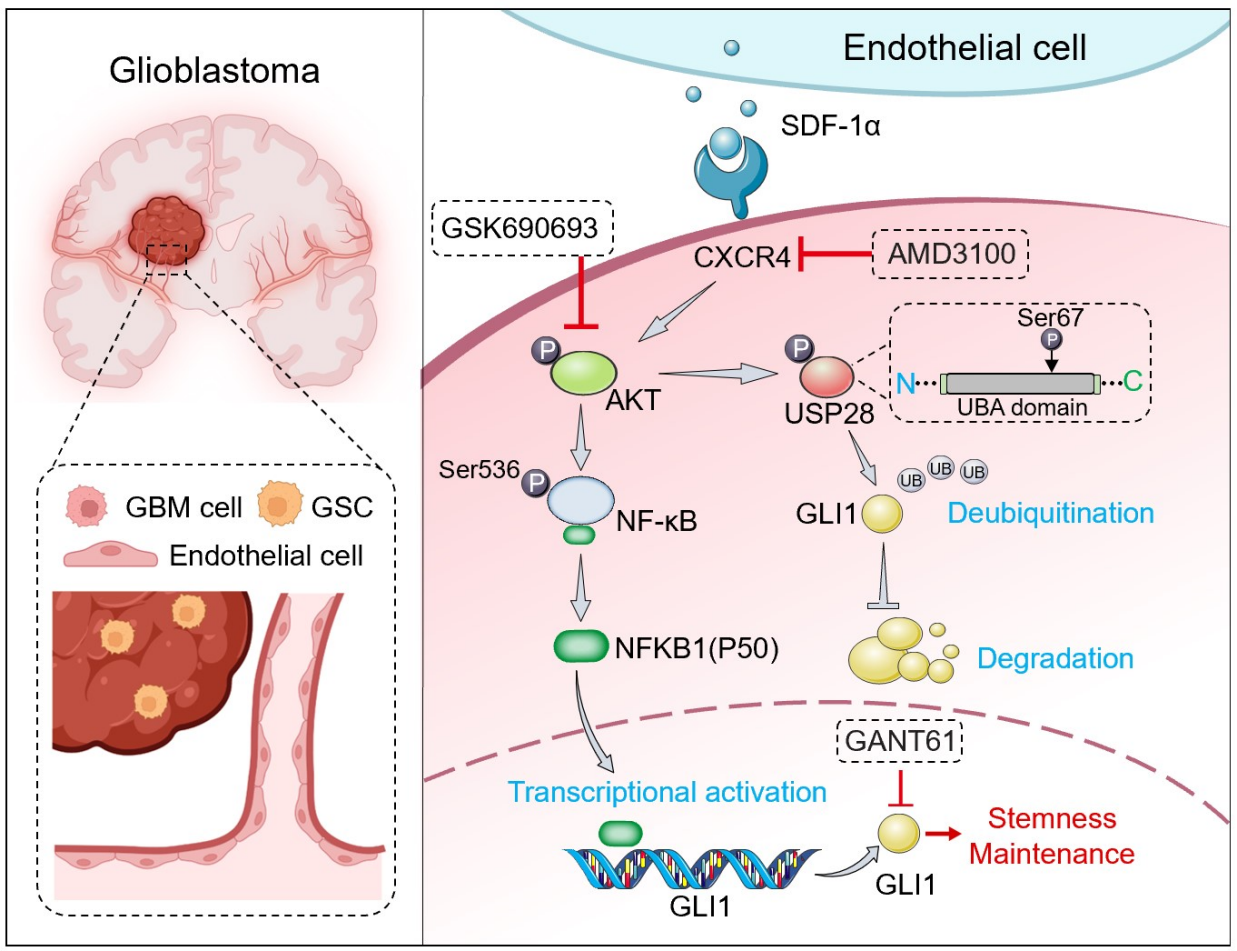
** Graphical abstract.** SDF-1α, secreted by ECs, activates the CXCR4-mediated AKT/NF-κB signaling pathway. This activation results in the direct transcriptional activation of GLI1 by the p50 subunit within the promoter region, thereby establishing a link between the NF-κB and Hedgehog signaling pathways. Furthermore, SDF-1α influences the turnover of GLI1 protein in GBM cells by modulating GLI1-associated polyubiquitin chains through the phosphorylation of the deubiquitinase USP28 at serine 67. In conclusion, the SDF-1α-CXCR4-AKT/NF-κB signaling axis enhances the stemness-maintaining properties of GLI1 through both transcriptional regulation and protein quality control mechanisms. Additionally, the combination of the CXCR4 antagonist AMD3100 and the AKT inhibitor GSK690693 demonstrates a synergistic effect in inhibiting GBM cell progression both* in vitro* and *in vivo*.

AKT promotes the activation of the inhibitor κB (IkB) kinase complex (IKK) to facilitate NF-κB transduction. NF-κB acts as a transcription factor in the nucleus, regulating inflammatory processes and innate immunity, both of which are crucial in tumorigenesis 32. Among the NF-κB protein family members, including RelA, RelB, c-Rel, p50, and p52, p50 (NFKB1) has been identified as a significant tumor-promoting factor in cancer proliferation and metastasis, correlating with poor patient survival 33,34. Due to the limited research but substantial therapeutic potential, our study focuses on characterizing p50 in GBM. Through validation studies, we discovered that p50 directly activates the transcription of GLI1 in the promoter region, bridging NF-κB and Hedgehog signaling pathways. PTMs of proteins are crucial for various cellular processes. Our investigation identified GLI1 as a substrate of the deubiquitinase USP28 in GBM and revealed a novel mechanism by which SDF-1α regulates GLI1 protein homeostasis. This regulation occurs through the phosphorylation of USP28 at serine 67, enhancing its deubiquitinating enzymatic activity and preventing the ubiquitin-proteasome degradation of GLI1. This finding provides new insights into the regulation of stemness (Figure 7). Within the tumor mass, varying levels of oxygen and nutrients from blood supply, along with exposure to angiocrine factors, lead to different metabolic zones in tumor cells, resulting in diverse phenotypic characteristics 35. While traditional *ex vivo* models cannot fully replicate the complex interactions in the TME, spatial transcriptomics technologies like laser capture microdissection, 10X Visium, and Nanostring GeoMX have limitations in capturing single-cell transcripts due to their relatively low resolution 36. To address this, we utilized an integrative approach inspired by the work of the Kumar group, focusing on the proximity of glioma cells to blood vessels. By perfusing tumor-bearing mice with Hoechst 33342, we classified the zonation of glioma cells around blood vessels based on the intensity of labeling 37. Our findings revealed active Hedgehog signaling in areas near blood vessels, which correlated with the spatial distribution of SDF-1α/CXCR4. Knocking down GLI1 reduced VEGF production by GSCs, thereby impacting the IGF-mediated angiogenic effects *in vitro* 38. This suggests a bidirectional regulation of GSC-vascular interactions by Hedgehog signaling. Further investigation is needed to elucidate how GLI1 regulates the secretome to drive the angiogenic phenotype of GSCs.

In conclusion, our study uncovered a previously unrecognized graded signaling axis between ECs and tumor cells, which facilitated the acquisition of stemness by non-GSCs. The stemness traits of non-GSCs were induced by ECs-derived SDF-1α through both transcriptional and post-translational regulation of GLI1. The combined treatment with a specific CXCR4 antagonist, AMD3100, and an AKT inhibitor, GSK690693, synergistically impeded the progression of GBM cells, suggesting novel potential therapeutic strategies for this cancer.

## Materials and Methods

### Cell culture

The LN229 human GBM cell line (ATCC-CRL-2611) was previously authenticated 39. The primary GBM cell line, GBM1, was established from tumor specimens obtained from GBM patients at TMMU 40. Glioma cells were isolated from GBM tumors by collecting resection tissues, which were then cut into small pieces and isolated using the Papain Dissociation System (Worthington Biochemical, Lakewood, NJ, USA) according to the manufacturer's instructions. LN229, GBM1, and human brain microvascular endothelial cells (hBMECs) were cultured in Dulbecco's Modified Eagle Medium (DMEM, Gibco, C11995500BT, USA) supplemented with 10% fetal bovine serum (FBS) (Gibco, a31608-02, USA) and 1% penicillin/streptomycin (Beyotime, C0222, China). For neurosphere formation, GBM cells were initially cultured in serum-free DMEM/F12 supplemented with 20 ng/mL basic fibroblast growth factor (bFGF, Peprotech, 100-18b), 20 ng/mL epidermal growth factor (EGF, Peprotech, AF-100-15), and 20 μL/mL B27 supplement (Gibco, 17504044, USA). To perform the limiting dilution sphere-forming assay, we employed a dual-compartment system (Transwell system) 41 to accommodate the distinct media requirements of GBM neurospheres (serum-free/stem cell conditions) and hBMECs (serum-containing media). Briefly, hBMECs were seeded in the upper chamber (0.8 μm pore size, #3428, Corning, USA), while GBM cells were placed in the lower chamber. GSC-like neurospheres were collected for serial passage after a 3-day cultivation in serum-free DMEM/F12 and were digested using papain (Gibco, 88280, USA). All cell lines were maintained at 37 °C in a humidified atmosphere with 5% CO_2_, and hypoxic culture was conducted under conditions of 1% O_2_, 94% N_2_, and 5% CO_2_ in an oxygen-adjustable incubator.

### Limiting dilution assay

In the limiting dilution assay, GBM cells were collected and plated in 96-well plates containing serum-free DMEM/F12 culture medium supplemented with 20 ng/mL bFGF, 20 ng/mL EGF, and 20 μL/mL B27. Cells were seeded at densities of 5, 10, 20, 40, 80, 160, and 200 cells per well, with 10 replicates for each density. After 14 days, the efficiency of neurosphere formation was assessed using extreme limiting dilution analysis, following established protocols 42.

### Quantitative reverse transcription PCR (qRT-PCR)

The total RNA from glioma cells was extracted according to the manufacturer's protocol, using the RNA Fast 2000 Reagent (Fastagen, China). The purified RNA was then reverse-transcribed with oligo-dT and random primers using the BcaBest RNA PCR kit (TaKaRa, RR023B, Japan). Subsequently, qRT-PCR was conducted on an iQ5 Multicolor Real-Time PCR Detection System (Bio-Rad) with a Real-time PCR Master Mix (SYBR Green, RR064B, Japan). The primer sequences are listed in Table S1.

### Immunofluorescence staining

Immunofluorescence staining of formaldehyde-fixed tissues involved a cryoprotection step using 15% sucrose in 1× Phosphate Buffer Saline (PBS) at 4 °C until the tissues sank. This was followed by a transfer to 30% sucrose in 1× PBS at 4 °C until they sank again. After removing the excess liquid, the tissues were embedded in OCT compound and sectioned into 6 µm-thick slices. The tissue or cell slices were then blocked for 30 minutes with goat serum, followed by incubation with primary antibodies overnight at 4 °C after dilution in 5% BSA. The primary antibodies used were CD133 (1:50, CST, #64326, USA) and αSMA (1:100, Abcam, ab7817, USA). After three washes, the cells were stained with secondary fluorescently-labeled antibodies (1:200, Proteintech, SA00013-2, USA) for 1 hour at room temperature. Following this, the samples were washed three times with PBS before staining with DAPI. Observations and photography were conducted using optical or confocal microscopes (Leica, DMi8, Germany).

### Western blotting

The western blotting assay was conducted according to previously established methods 20. The primary antibodies used in this study were as follows: β-actin (CST, #3700, USA), SOX2 (CST, #3579, USA), OCT4 (CST, #2750, USA), NANOG (CST, #4903, USA), CD133 (CST, #64326, USA), CXCR4 (Abcam, ab124824, USA), GLI1 (CST, #2643, USA), NOTCH1 (CST, #3608, USA), β-catenin (CST, 8480, USA), GAPDH (CST, #3683, USA), Lamin B1 (CST, #13435, USA), UB (CST, #58395, USA), p-AKT (CST, #4060, USA), AKT (CST, #2920, USA), and phospho-NFκB p65 (Ser536) (CST, #3033, USA), USP7 (Abcam, ab108931, UK), USP19 (Abcam, ab189518, UK), USP28 (Abcam, ab126604, UK), Phospho-USP28 (Ser67) (Invitrogen, PA5-64727, USA), Phospho-USP28 (Ser714) (Affinity, AF8333, USA).

### Immunohistochemistry (IHC)

IHC staining of CD31, SOX2, and CD133 in glioblastoma (GBM) xenografts was conducted using the Dako REAL EnVision Detection System, following the manufacturer's protocol (DAKO, Carpinteria, CA, USA). Primary antibodies, including anti-CD31 (Abcam, ab28364, UK), anti-SOX2 (Abcam, ab92494, UK), and anti-CD133 (Abcam, ab278053, UK), were utilized at a concentration of 10 µg/mL. All images were captured using a Leica DM4000B microscope, which was equipped with a QImaging EXiAqua camera (QImaging, Surrey, BC, Canada).

### Co-immunoprecipitation

GBM cells were lysed on ice for 40 minutes using RIPA lysis buffer, which was supplemented with a protease inhibitor cocktail. The supernatant proteins were collected and incubated with the specified primary antibodies or their corresponding isotype IgG (CST, #3900, USA). Subsequently, the proteins were analyzed utilizing a Pierce™ Co-Immunoprecipitation Kit (Thermo Scientific, 26149, USA) in accordance with the manufacturer's protocol.

### FACS analysis and sorting

Neurospheres were dissociated into single cells and subsequently incubated with SOX2 antibody (Abcam, ab279687, UK) and CD133 APC-conjugated antibody (Miltenyi, 130-090-826, Germany) for 30 minutes at 4 °C. To reduce potential non-specific antibody binding caused by IgG receptors and to exclude dead cells, Mouse FC Block and 7-amino-actinomycin D staining (BD Falcon, USA) were utilized. Isotype controls served as negative controls to assess non-specific background staining across all experiments. Following three washes with PBS, the samples were analyzed using flow cytometry (FACS Aria II, BD Falcon, USA). Cell sorting was carried out using the same FACS equipment (FACS Aria II, BD Falcon, USA).

### Transfection and dual-luciferase reporter assay

GBM cells were co-transfected at 70-80% confluency with 3 μg of GLI1 promoter reporter vector and 3 μg of Renilla control vector (GeneChem, Shanghai, China) at a concentration of 500 ng using Lipofectamine 3000 (Invitrogen, USA) for 24 hours. Subsequently, the GBM cells were transfected with either an NFKB1 expression vector or an empty vector (GeneChem, Shanghai, China). The fluorescent signal was assessed 48 hours post-transfection utilizing the Dual-Luciferase Reporter Assay System (Promega, Madison, WI, USA) with three biological replicates. The relative luciferase activity was determined by normalizing the measured values to the firefly luciferase activity.

### Clone formation assay

Cells were plated on 6-well plates at a density of 500 cells per well. After 14 days, the cancer cells were treated with 0.5% crystal violet, and a colony was defined as containing at least 50 cells. The clonal efficiency was then compared across different groups.

### CCK-8 assay

A total of 1.5×10^3^ GBM cells were seeded in each well of 96-well plates and then assessed using CCK-8 reagent from Dojindo, Kumamoto, Japan. The optical density (OD) value was measured at 450 nm using a fluoroanalyzer (Floskan Ascent, Waltham, MA, USA) over a period of 6 consecutive days.

### Transwell migration and invasion assay

A Transwell membrane (Millipore in Billerica, USA) was coated with Matrigel (BD Biosciences) at a concentration of 1 mg/mL. Subsequently, 500 μL of DMEM supplemented with 10% FBS was added to the lower chambers, while GBM cells were seeded in the upper chambers at a density of 2 × 10^4^ cells in 200 μL of DMEM. The cells were incubated at 37 °C with 5% CO_2_ for 24 hours. Following incubation, the filter membranes were fixed with 4% formaldehyde for 15 minutes. The cells in the upper chamber were stained with crystal violet for an additional 15 minutes. Cell migration was evaluated using a Matrigel-uncoated Transwell membrane, with the same protocol as the invasion assay.

### ELISAs

The levels of soluble stromal cell-derived factor-1α (SDF-1α) protein in GBM cells, ECs, and culture supernatants were measured using ELISA kits (Elabscience, E-EL-H0052c, China) following the manufacturer's instructions.

### Chromatin immunoprecipitation (CHIP)

CHIP assays were conducted using the SimpleChIP® Enzymatic Chromatin IP Kit (CST, #9003, USA). Cells transfected with the NFKB1 plasmid for 48 hours were subjected to CHIP analysis using an anti-NFKB1 antibody alongside a normal IgG antibody. The precipitated DNA was amplified *via* PCR using specific primers. The resulting PCR products were analyzed through agarose gel electrophoresis and visualized with ethidium bromide under ultraviolet light. Subsequently, the purified DNA was analyzed using real-time PCR with the SYBR Green mix (Bio-Rad, USA) on the MyIQ machine (Bio-Rad, USA).

### Construction of expression plasmids

Flag-tagged USP7, USP19, and USP28 were obtained from GeneChem (Shanghai, China). Various truncated USP28 mutants were amplified *via* PCR from the USP28 cDNA plasmid and subsequently cloned into the pcDNA3.1+/Zeo vector. The USP28 mutant containing the S-to-A substitution (S67A) was created using the FastMutagenesis System (TransGen Biotech, Beijing, China), with pCMV6-Flag-USP28 serving as the template. The identity of each plasmid was verified through DNA sequencing.

### RNA interference assays

To inhibit AKT, GLI1, and NFKB1 in glioblastoma (GBM) cells, short hairpin RNA (shRNA) and a non-targeting scrambled RNA control (shCtrl) were constructed using lentiviral vectors obtained from HANBIO (Shanghai, China). Stably transfected cells were selected by culturing in the presence of 2.5 µg/mL puromycin.

### *In vitro* kinase assay

*In vitro* kinase assays were conducted by adding purified active kinases and substrates to a final volume of 40 μL, which contained a kinase buffer composed of 20 mM Tris-HCl (pH 7.5), 5 mM MgCl_2_, 1 mM DTT, and 150 mM KCl, along with 5 μM ATP. The reactions were incubated at 30 °C for 30 minutes and subsequently terminated by boiling in Laemmli buffer. The phosphorylated proteins were analyzed using 10% SDS-PAGE.

### *In vivo* ubiquitination assay

To investigate the ubiquitination of GLI1, GBM cells were transfected with HA-Ub (ubiquitin) along with specified vectors for a duration of 24 hours. Whole-cell lysates were obtained using a buffer containing 50 mM Tris-HCl (pH 7.5), 0.5 mM EDTA, 1 mM DTT, 1% SDS, and a protease inhibitor, followed by incubation at 95 °C for 10 minutes. Subsequently, 900 μL of Tris-HCl buffer (50 mM, pH 7.5) was added, and the solution was sonicated, and centrifuged. The resulting cell lysates were then subjected to immunoprecipitation.

### Data processing and collation

This study involved the collection and analysis of two distinct public datasets on glioma, comprising single-cell RNA sequencing (scRNA-seq) data and bulk RNA sequencing data, both matched with comprehensive clinical information. The scRNA-seq dataset, GSE182109 (https://www.ncbi.nlm.nih.gov/geo/query/acc.cgi?acc=GSE182109), included 44 samples from 18 patients with various types of gliomas (LGG = 3, rGBM = 18, ndGBM = 23), sourced from the GEO database. Furthermore, transcriptomic data and clinical information for glioma patients were obtained from the Chinese Glioma Genome Atlas database (CGGA) (https://www.cgga.org.cn/download.jsp), encompassing 693 patients (WHO II = 188, WHO III = 255, WHO IV = 259). After excluding patients lacking survival data, a total of 624 tumor patients were included in the analysis.

### Single-cell data processing

The Seurat software (version 4.4.0) was utilized to preprocess the glioma single-cell RNA sequencing (scRNA-seq) dataset GSE182109, obtained from the GEO database (https://www.ncbi.nlm.nih.gov/geo/query/acc.cgi?acc=GSE182109). Initially, quality control measures were implemented to exclude clusters containing fewer than five cells and individual cells detected in fewer than 300 cells. High-quality cells were defined according to specific criteria: nFeature_RNA > 200, nFeature_RNA < 6000, percent_RP < 40, percent_MT < 20, percent_HB < 0.2, and nCount_RNA < 25000. Batch effects were mitigated using Harmony (version 2.1.0). After normalization via the LogNormalize function, principal component analysis was performed on the top 2000 highly variable genes using the RunPCA function. The top 10 principal components and a reduction factor of 0.8 were selected for Uniform Manifold Approximation and Projection (UMAP) and t-distributed Stochastic Neighbor Embedding (tSNE) analyses. The AddModuleScore function was employed to perform module scoring of glioma stem cells (GSCs). Marker genes for each cluster were identified using the FindAllMarkers function. Cell subgroups were annotated using SingleR (version 2.4.1), CellMarker (http://xteam.xbio.top/CellMarker/), and PanglaoDB (https://panglaodb.se/index.html).

### Cell-cell communication analysis

The tumor microenvironment represents a complex ecosystem characterized by extensive cellular interactions, primarily mediated by receptors and ligands on the cell surface that facilitate intercellular signaling. In this study, we employed CellChat (version 1.6.1) to investigate the communication patterns between cancer stem cells and endothelial cells in glioma. CellChat utilizes network analysis and pattern recognition techniques to predict the primary signaling interactions among cells while also providing robust data visualization capabilities.

### The construction of the endothelial cell scoring model

This study identified 60 markers related to endothelial cells through a comprehensive literature review. These markers were then combined with the top 100 endothelial cell genes derived from single-cell transcriptome analysis to establish a final gene set. A scoring model for endothelial cells was developed using Gene Set Variation Analysis (GSVA; version 1.50.5) with bulk RNA sequencing data from the CGGA 693 dataset (https://www.cgga.org.cn/). Spearman's correlation was utilized to analyze the relationship between the identified genes and the endothelial cell score. Furthermore, survival analysis was performed using the 'survival' package (version 3.5-5) and the 'survminer' package (version 0.4.9) to categorize significant molecules and sample scores for subsequent analysis.

### Microarray analysis

Transcriptome profiling using microarrays was conducted by accessing data from the Gene Expression Omnibus (GEO) portal with accession number GSE75003 (https://www.ncbi.nlm.nih.gov/gds/?term=GSE75003) and the ArrayExpress repository with accession number E-MTAB-6882 (https://www.ebi.ac.uk/biostudies/arrayexpress/studies/E-MTAB-6882). In the E-MTAB-6882 dataset, *Ensembl IDs* were transformed into *Gene Symbols* to extract expression levels of well-recognized GSC markers, including CD133, CD15, SOX2, Nestin, and NANOG from the data matrix. Enrichment scores for the aforementioned gene collection and stemness signaling pathways, including Notch, Hedgehog, and Wnt-β-catenin, were calculated across far and close groups using the *ssGSEA* function from the *GSVA* R package; the scaled scoring matrix was visualized using the *pheatmap* (version 1.0.12) package. For the GSE75003 dataset, the chip file in Affy CEL format was imported into R using the *ReadAffy* function from the *affy* (version 1.80.0) R package. The *gcrma* (version 2.74.0) package, which includes background correction and quantile normalization processes, was used to generate probe set expression values. *Probe IDs* were converted into *Gene Symbols* using the *hgu133plus2*.db (version 3.13.0) and *annotate* (version 1.80.0) packages, while the *do_DEG_2groups* function from the *humanid* package was utilized to identify differentially expressed genes (DEGs). To investigate the impact of NF-κB signaling on the Hedgehog-GLI axis, Gene Set Enrichment Analysis (GSEA) was performed using the *GSEA* function from the *clusterProfiler* (version 4.10.1) R package, with results plotted using the *enrichplot* (version 1.22.0) R package. All gene sets were obtained from the Molecular Signatures Database (MsigDB, https://www.gsea-msigdb.org/gsea/msigdb) and input into R using the *msigdbr* (version 7.5.1) function.

### Spatial transcriptomic analysis through IvyGAP

Colorimetric data for CD15 (FUT4) at the cellular resolution was obtained through the evaluation of In Situ Hybridization (ISH) signals in GBM tissue sections, which were anatomically annotated by the Ivy Glioblastoma Atlas Project (IvyGAP, http://glioblastoma.alleninstitute.org/). The spatial dissection of SDF-1 and CXCR4 expression patterns was conducted using an online analysis and visualization system.

### Xenografts model

The animal experiments conducted in this study received approval from the Animal Care and Use Committee of PLA General Hospital and adhered to the guidelines established by the National Institutes of Health. Female NOD-SCID mice, aged 4 to 6 weeks and weighing between 18 and 20 grams, were anesthetized for the procedure. GBM cells were injected intracranially either alone or in a mixture with tumor and endothelial cells at a 1:1 ratio, with a cell density of 2 × 10^5 cells in 5 µL of PBS. The injection site was located 2 mm lateral and 1 mm rostral to the bregma, and 3 mm deep from the skull surface. Drug treatments commenced on day 5 post-implantation and were categorized into three groups: DMSO (1%/day), AMD3100 (2 mg/kg/day), GSK690693 (25 mg/kg/day), XAV-939 (15 mg/kg/day), and GANT61 (20 mg/kg/day), all sourced from Selleck in the USA. Approximately on days 5, 18, and 20 post-injection, luciferin was administered intraperitoneally to track tumor cells in vivo. The mice were subsequently anesthetized, and tumor burden was assessed using bioluminescence imaging with the In Vivo Imaging System (IVIS) Lumina from Perkin-Elmer in Waltham, MA. Image analysis was conducted using Living Image software.

### Statistical analysis

Statistical analyses in bioinformatics were conducted using R software (version 4.3.0). A significance level of P < 0.05 was established, unless otherwise specified (*, *P* < 0.05; **, *P* < 0.01; ***, *P* < 0.001; n.s., not significant). ImageJ software was employed to quantify the gray values of blot bands. Data visualization and statistical analyses were performed using Prism software (GraphPad Software, San Diego, CA, USA). Two-tailed Student's t-tests were utilized for single comparisons of normally distributed data, while *ANOVA* with Bonferroni post hoc tests was applied for multiple comparisons, unless stated otherwise. For non-normally distributed data, the Mann-Whitney U test was used. Additionally, Pearson correlation analysis was conducted to assess the relationship between two continuous variables, and survival analysis was performed using the Kaplan-Meier method.

## Supplementary Material

Supplementary figures and table.

## References

[1] Eyupoglu IY, Buchfelder M, Savaskan NE (2013). Surgical resection of malignant gliomas-role in optimizing patient outcome. Nat Rev Neurol.

[2] Omuro A, DeAngelis LM (2013). Glioblastoma and other malignant gliomas: a clinical review. Jama-J Am Med Assoc.

[3] Suva ML, Tirosh I (2020). The Glioma Stem Cell Model in the Era of Single-Cell Genomics. Cancer Cell.

[4] Gimple RC, Bhargava S, Dixit D, Rich JN (2019). Glioblastoma stem cells: lessons from the tumor hierarchy in a lethal cancer. Gene Dev.

[5] Lathia JD, Mack SC, Mulkearns-Hubert EE, Valentim CLL, Rich JN (2015). Cancer stem cells in glioblastoma. Gene Dev.

[6] Gilbertson RJ, Rich JN (2007). Making a tumour's bed: glioblastoma stem cells and the vascular niche. Nat Rev Cancer.

[7] Prager BC, Bhargava S, Mahadev V, Hubert CG, Rich JN (2020). Glioblastoma Stem Cells: Driving Resilience through Chaos. Trends Cancer.

[8] Lee JH, Lee JE, Kahng JY, Kim SH, Park JS, Yoon SJ (2018). Human glioblastoma arises from subventricular zone cells with low-level driver mutations. Nature.

[9] Matarredona ER, Pastor AM (2019). Neural Stem Cells of the Subventricular Zone as the Origin of Human Glioblastoma Stem Cells. Therapeutic Implications. Front Oncol.

[10] Safa AR, Saadatzadeh MR, Cohen-Gadol AA, Pollok KE, Bijangi-Vishehsaraei K (2015). Glioblastoma stem cells (GSCs) epigenetic plasticity and interconversion between differentiated non-GSCs and GSCs. Genes Dis.

[11] Jeon H, Sohn Y, Oh S, Kim S, Beck S, Kim S (2011). ID4 imparts chemoresistance and cancer stemness to glioma cells by derepressing miR-9*-mediated suppression of SOX2. Cancer Res.

[12] Schmid RS, Simon JM, Vitucci M, McNeill RS, Bash RE, Werneke AM (2016). Core pathway mutations induce de-differentiation of murine astrocytes into glioblastoma stem cells that are sensitive to radiation but resistant to temozolomide. Neuro Oncol.

[13] Schonberg DL, Lubelski D, Miller TE, Rich JN (2014). Brain tumor stem cells: Molecular characteristics and their impact on therapy. Mol Aspects Med.

[14] Mondal S, Bhattacharya K, Mandal C (2018). Nutritional stress reprograms dedifferention in glioblastoma multiforme driven by PTEN/Wnt/Hedgehog axis: a stochastic model of cancer stem cells. Cell Death Discov.

[15] Carlson JC, Cantu Gutierrez M, Lozzi B, Huang-Hobbs E, Turner WD, Tepe B (2021). Identification of diverse tumor endothelial cell populations in malignant glioma. Neuro Oncol.

[16] Mei X, Chen Y, Chen F, Xi S, Chen Z (2017). Glioblastoma stem cell differentiation into endothelial cells evidenced through live-cell imaging. Neuro Oncol.

[17] Jiang Z, Zhou W, Guan S, Wang J, Liang Y (2013). Contribution of SDF-1alpha/CXCR4 signaling to brain development and glioma progression. Neurosignals.

[18] Weller M, Wen PY, Chang SM, Dirven L, Lim M, Monje M (2024). Glioma. Nat Rev Dis Primers.

[19] Lei Y, Tang R, Xu J, Wang W, Zhang B, Liu J (2021). Applications of single-cell sequencing in cancer research: progress and perspectives. J Hematol Oncol.

[20] Lapidot T, Kollet O (2002). The essential roles of the chemokine SDF-1 and its receptor CXCR4 in human stem cell homing and repopulation of transplanted immune-deficient NOD/SCID and NOD/SCID/B2m(null) mice. Leukemia.

[21] Liu Z, Tian R, Li Y, Zhang L, Shao H, Yang C (2016). SDF-1alpha-induced dual pairs of E-selectin/ligand mediate endothelial progenitor cell homing to critical ischemia. Sci Rep-Uk.

[22] Jin F, Brockmeier U, Otterbach F, Metzen E (2012). New insight into the SDF-1/CXCR4 axis in a breast carcinoma model: hypoxia-induced endothelial SDF-1 and tumor cell CXCR4 are required for tumor cell intravasation. Mol Cancer Res.

[23] Zhou Y, Cao H, Li W, Zhao L (2018). The CXCL12 (SDF-1)/CXCR4 chemokine axis: Oncogenic properties, molecular targeting, and synthetic and natural product CXCR4 inhibitors for cancer therapy. Chin J Nat Medicines.

[24] McIntosh SZ, Maestas MM, Dobson JR, Quinn KE, Runyan CL, Ashley RL (2021). CXCR4 signaling at the fetal-maternal interface may drive inflammation and syncytia formation during ovine pregnancydagger. Biol Reprod.

[25] Ren X, Jiang M, Ding P, Zhang X, Zhou X, Shen J (2023). Ubiquitin-specific protease 28: the decipherment of its dual roles in cancer development. Exp Hematol Oncol.

[26] Piper K, DePledge L, Karsy M, Cobbs C (2021). Glioma Stem Cells as Immunotherapeutic Targets: Advancements and Challenges. Front Oncol.

[27] Zhou W, Chen C, Shi Y, Wu Q, Gimple RC, Fang X (2017). Targeting Glioma Stem Cell-Derived Pericytes Disrupts the Blood-Tumor Barrier and Improves Chemotherapeutic Efficacy. Cell Stem Cell.

[28] Wang D, Prager BC, Gimple RC, Aguilar B, Alizadeh D, Tang H (2021). CRISPR Screening of CAR T Cells and Cancer Stem Cells Reveals Critical Dependencies for Cell-Based Therapies. Cancer Discov.

[29] Fidler IJ, Poste G (2008). The "seed and soil" hypothesis revisited. Lancet Oncol.

[30] Couturier CP, Ayyadhury S, Le PU, Nadaf J, Monlong J, Riva G (2020). Single-cell RNA-seq reveals that glioblastoma recapitulates a normal neurodevelopmental hierarchy. Nat Commun.

[31] Jeon H, Jin X, Lee J, Oh S, Sohn Y, Park H (2008). Inhibitor of differentiation 4 drives brain tumor-initiating cell genesis through cyclin E and notch signaling. Gene Dev.

[32] Yang L, Hu X, Mo Y (2019). Acidosis promotes tumorigenesis by activating AKT/NF-kappaB signaling. Cancer Metast Rev.

[33] Porta C, Ippolito A, Consonni FM, Carraro L, Celesti G, Correale C (2018). Protumor Steering of Cancer Inflammation by p50 NF-kappaB Enhances Colorectal Cancer Progression. Cancer Immunol Res.

[34] Dai M, Hu S, Liu C, Jiang L, Yu W, Li Z (2019). BPTF cooperates with p50 NF-kappaB to promote COX-2 expression and tumor cell growth in lung cancer. Am J Transl Res.

[35] Guillaumond F, Leca J, Olivares O, Lavaut M, Vidal N, Berthezene P (2013). Strengthened glycolysis under hypoxia supports tumor symbiosis and hexosamine biosynthesis in pancreatic adenocarcinoma. P Natl Acad Sci Usa.

[36] Moses L, Pachter L (2022). Museum of spatial transcriptomics. Nat Methods.

[37] Kumar S, Sharife H, Kreisel T, Mogilevsky M, Bar-Lev L, Grunewald M (2019). Intra-Tumoral Metabolic Zonation and Resultant Phenotypic Diversification Are Dictated by Blood Vessel Proximity. Cell Metab.

[38] Hsieh A, Ellsworth R, Hsieh D (2011). Hedgehog/GLI1 regulates IGF dependent malignant behaviors in glioma stem cells. J Cell Physiol.

[39] Li Y, He Z, Zhang X, Liu Q, Chen C, Zhu Z (2018). Stanniocalcin-1 augments stem-like traits of glioblastoma cells through binding and activating NOTCH1. Cancer Lett.

[40] Yuan Y, Wang L, Zhao X, Wang J, Zhang M, Ma Q (2022). The E3 ubiquitin ligase HUWE1 acts through the N-Myc-DLL1-NOTCH1 signaling axis to suppress glioblastoma progression. Cancer Commun.

[41] Zhang X, Yang K, Chen C, He Z, Wang Q, Feng H (2021). Pericytes augment glioblastoma cell resistance to temozolomide through CCL5-CCR5 paracrine signaling. Cell Res.

[42] Yuan Y, Yan Z, Miao J, Cai R, Zhang M, Wang Y (2019). Autofluorescence of NADH is a new biomarker for sorting and characterizing cancer stem cells in human glioma. Stem Cell Res Ther.

