# Supplementary material for: Endothelial cell-derived SDF-1α elicits stemness traits of glioblastoma via dual-regulation of GLI1
Source: Theranostics. 2025 Sep 8;15(18):9819–37. doi: 10.7150/thno.108843 (PMC12486407; doi:10.7150/thno.108843)
Supplement: Supplementary file 1 — Supplementary figures and table. [file thnov15p9819s1.pdf]

Supplementary Information for

Endothelial cell-derived SDF-1α elicits stemness traits of glioblastoma *via* dual-regulation of GLI1

Ye Yuan[1†], Xudong Liu[2,5†], Liwen Kuang[2], Shixue Yang[1], Lihong Wang[3], Jiao Wang[3], Sen Wei[3], Zexuan Yan[3], Qinghua Ma[3], Juan Lei[1], Yu Zhou[1], Yu Chen[1], Jiongming Chen[2], Tao Luo[3], Kaidi Yang[4*], Mengsi Zhang[3*], Yongsheng Li[1*]

1.Department of Medical Oncology, Chongqing University Cancer Hospital, Chongqing, 400030, China.

2. School of Medicine, Chongqing University, Chongqing 400044, China.

3. Institute of Pathology and Southwest Cancer Center, Southwest Hospital, Third Military Medical University (Army Medical University), Chongqing 400038, China.

4. Department of Oncology, Hainan Hospital of Chinese People's Liberation Army General Hospital, Sanya, Hainan Province 572013, PR China.

5. Bioengineering College of Chongqing University, Chongqing, 400030, China.

Running title: Stemness traits regulation by endothelial cells

†These authors contributed equally to this work.

* Corresponding authors: lys@cqu.edu.cn (YL); mengsijane@163.com (MZ) ; lampirl@163.com (KY).

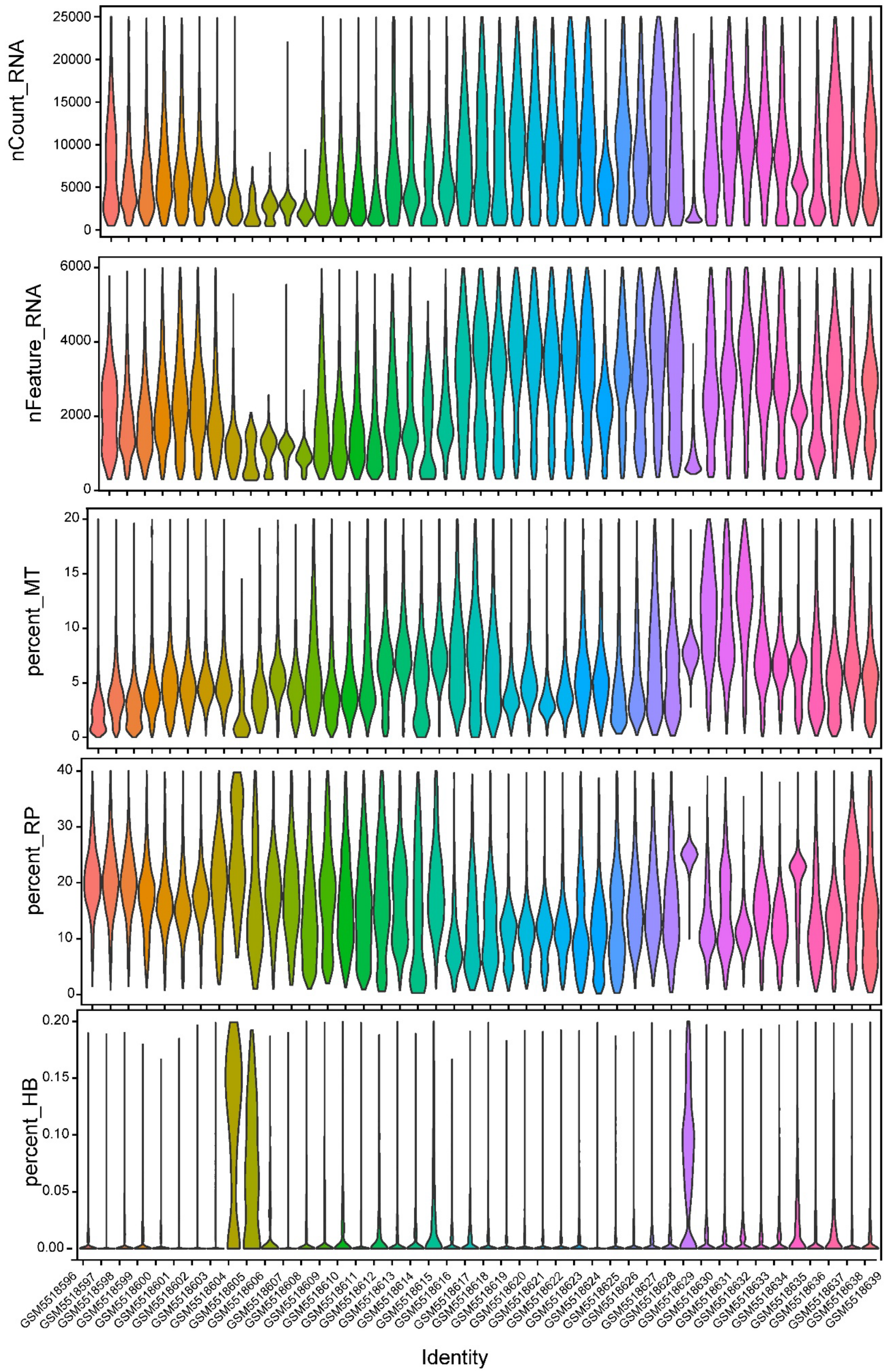

Figure S1 **Filtering of low-quality cells.**

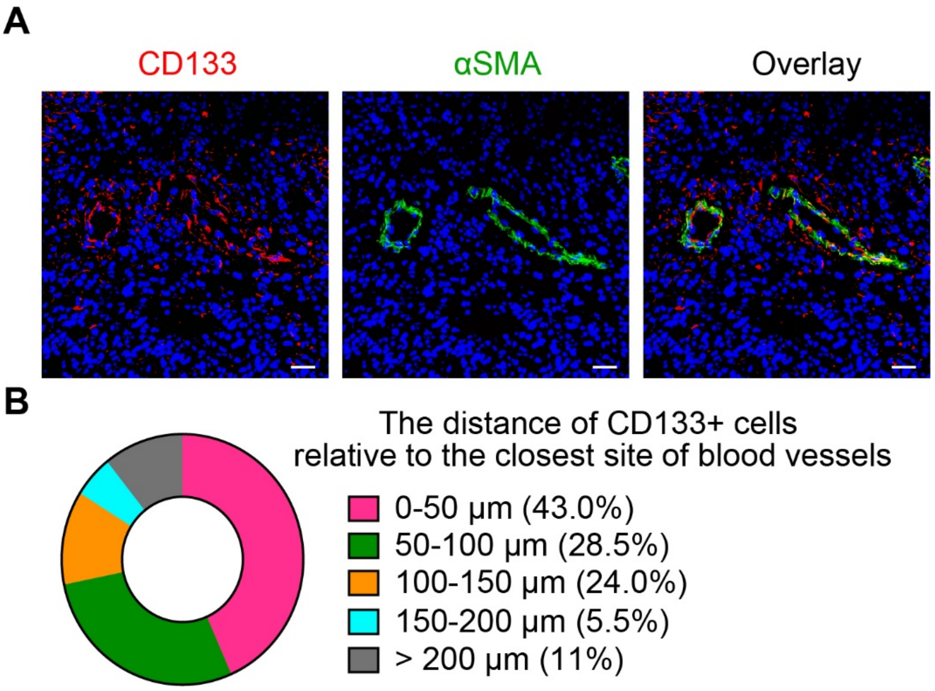

Figure S2 Spatial tissue localization of GSCs relative to microvessels.

(A) Immunofluorescent staining image illustrating co-localization of GSC marker CD133 (red) and vascular pericyte marker αSMA (green) in human GBM tissue. Scale bar = 50 μm. **(B)** The overall perivascular distribution of GSCs was defined by calculating the distances between $CD133^+$ cells and the closest sites in the blood vessel ring, data were collected from analyzing 200 GSCs.

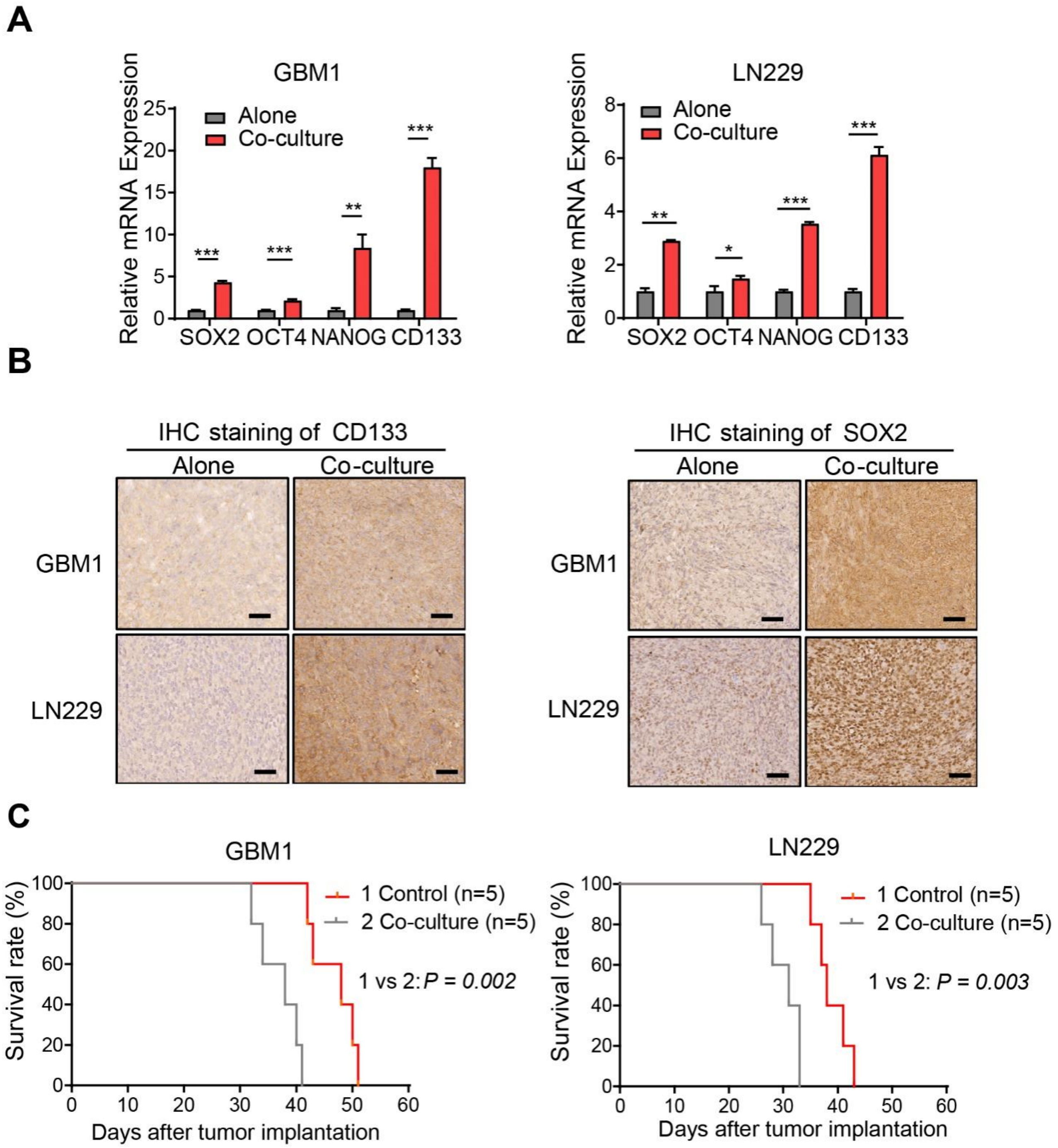

Figure S3 ECs induce the expression of stemness markers and traits in co-cultured non-GSC populations.

(A) qRT-PCR assay tested the expression of SOX2, OCT4, NANOG, and CD133 in ECs co-cultured and control GBM cells. **(B)** Representative images depicting IHC staining of CD133 and SOX2 in xenografts from mice bearing GBM cells, both co-cultured with ECs and in control conditions. Scale

bar = 40 um. **(C)** Kaplan-Meier survival analysis of the mice bearing xenograft tumors of GBM cells, comparing those with prior co-culture with ECs to those with GBM cells alone. Data are means ± SD. *, $P < 0.05$; **, $P < 0.01$; ***, $P < 0.001$.

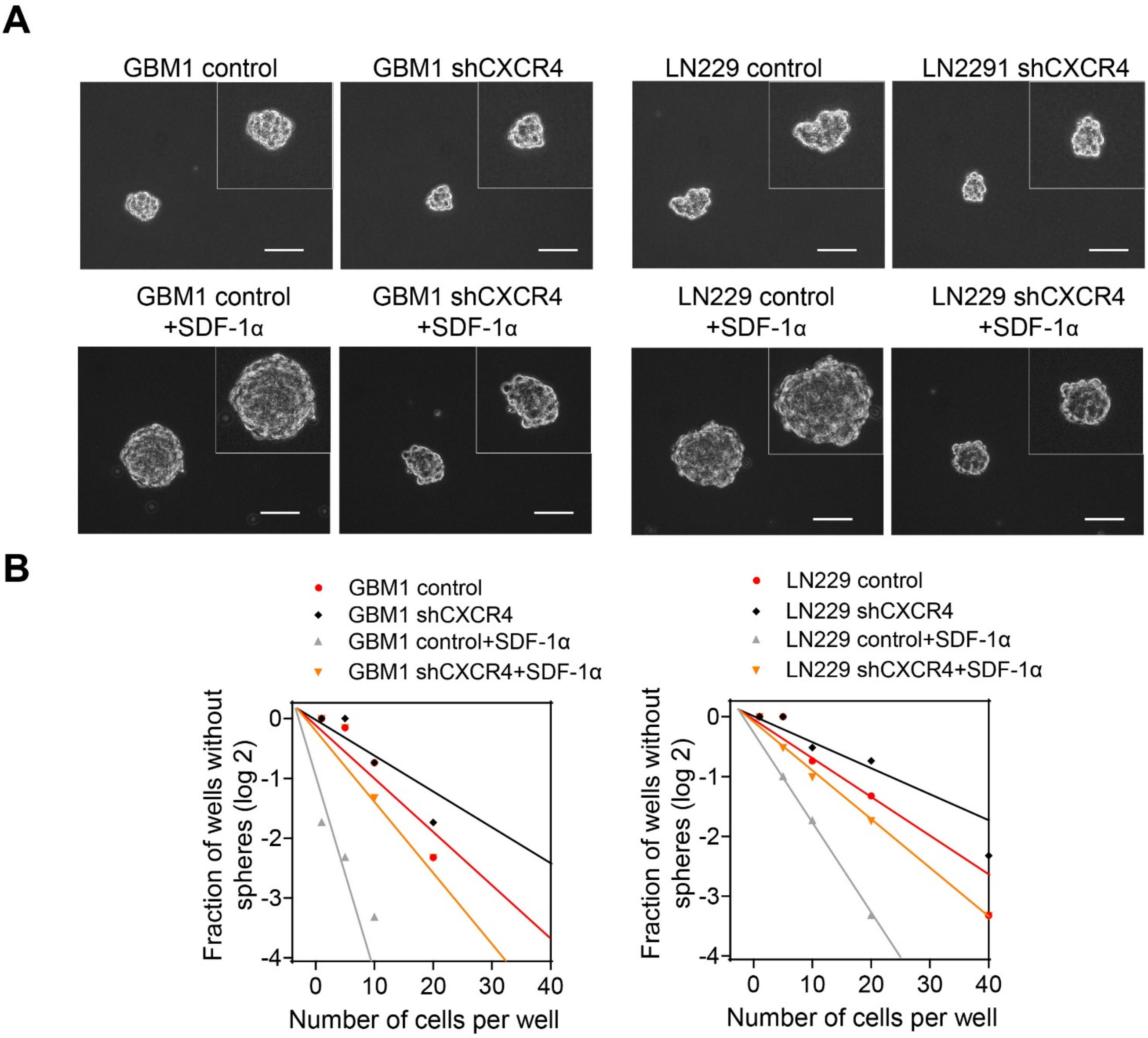

Figure S4 Disrupting CXCR4 attenuated the SDF-1α dependent stemness in GBM cells.

(A-B) Limiting dilution sphere-forming assays conducted on GBM1 **(A)** and LN229 **(B)** cells treated with either SDF-1α, shCXCR4, or a combination of both, along with representative images of the resulting tumor spheres.

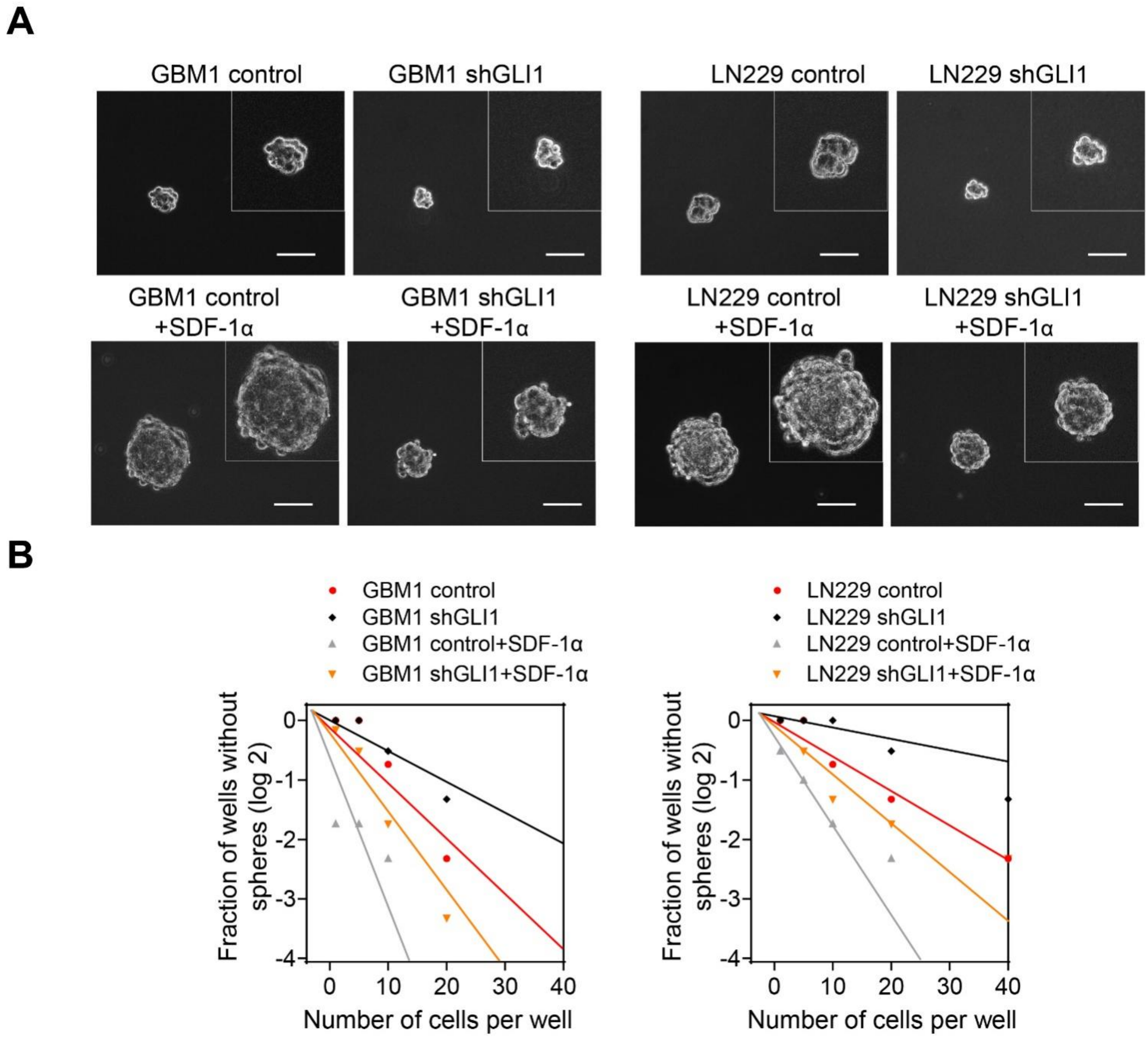

Figure S5 Disrupting GLI1 attenuates SDF-1α dependent stemness in GBM cells.

(A-B) Limiting dilution sphere-forming assays conducted on GBM1 **(A)** and LN229 **(B)** cells treated with either SDF-1α, shGLI1, or a combination of both, along with representative images of the resulting tumor spheres.

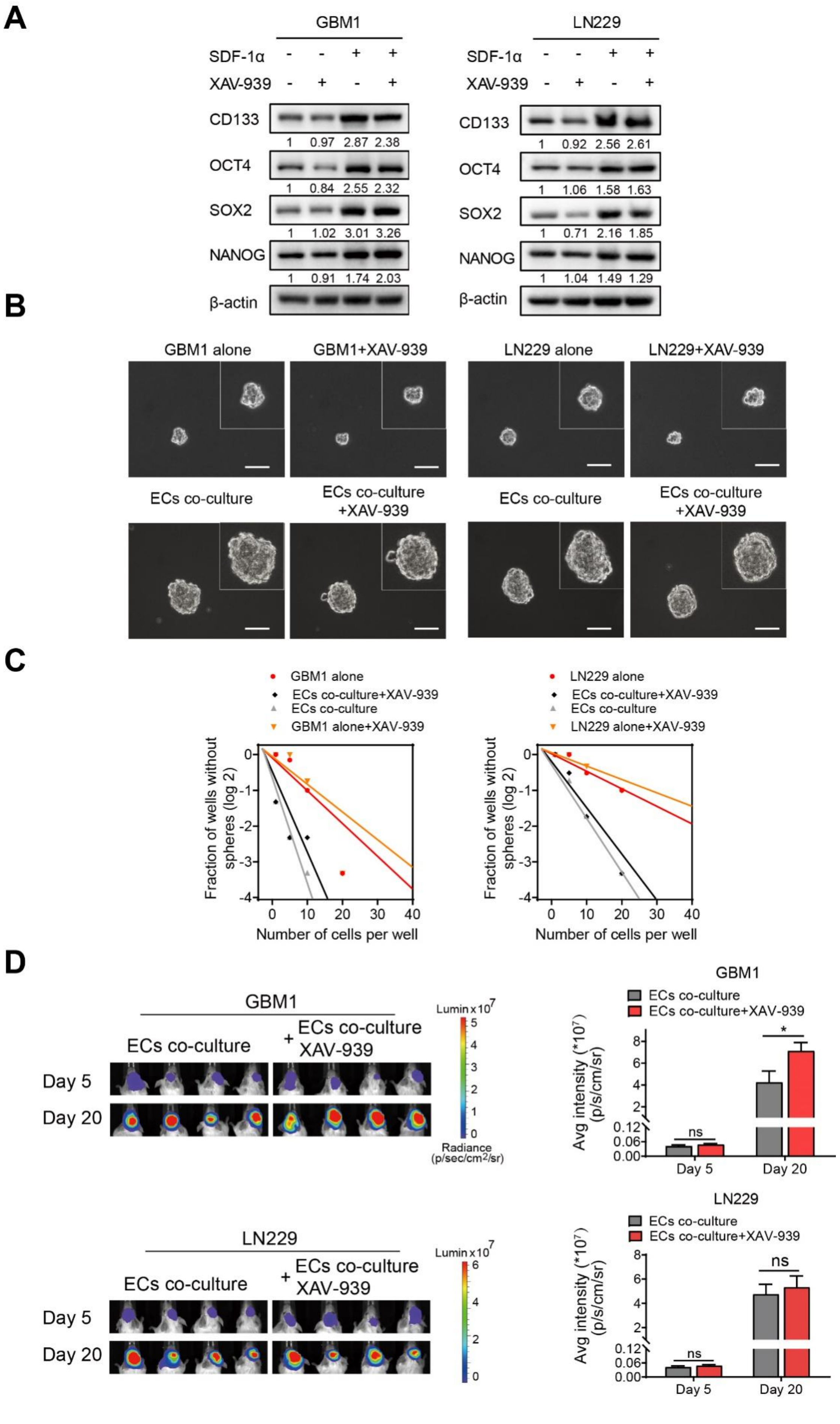
A
GBM1
SDF-1α - - + +
XAV-939 - + - +
CD133
1 0.97 2.87 2.38
OCT4
1 0.84 2.55 2.32
SOX2
1 1.02 3.01 3.26
NANOG
1 0.91 1.74 2.03
β-actin
LN229
SDF-1α - - + +
XAV-939 - + - +
CD133
1 0.92 2.56 2.61
OCT4
1 1.06 1.58 1.63
SOX2
1 0.71 2.16 1.85
NANOG
1 1.04 1.49 1.29
β-actin
B
GBM1 alone
GBM1+XAV-939
LN229 alone
LN229+XAV-939
ECs co-culture
ECs co-culture +XAV-939
ECs co-culture
ECs co-culture +XAV-939
C
GBM1 alone
ECs co-culture+XAV-939
ECs co-culture
GBM1 alone+XAV-939
LN229 alone
ECs co-culture+XAV-939
ECs co-culture
LN229 alone+XAV-939
Fraction of wells without spheres (log 2)
Number of cells per well
D
GBM1
ECs co-culture
ECs co-culture + XAV-939
Day 5
Day 20
Lumin x10^7
Radiance (p/sec/cm^2/sr)
GBM1
ECs co-culture
ECs co-culture+XAV-939
Avg intensity (*10^7) (p/s/cm/sr)
*
ns
Day 5
Day 20
LN229
ECs co-culture
ECs co-culture + XAV-939
Day 5
Day 20
Lumin x10^7
LN229
ECs co-culture
ECs co-culture+XAV-939
Avg intensity (*10^7) (p/s/cm/sr)
ns
ns
Day 5
Day 20

Figure S6 XAV-939 demonstrated few or none inhibitory effect on SDF-1α dependent stemness maintaining in GBM cells.

(A) Western blot assay tested the expression of SOX2, OCT4, NANOG, and CD133 in GBM cells treated with SDF-1α/XAV-939 alone or together. **(B-C)** Limiting dilution sphere-forming assays were performed on ECs co-cultured with control GBM cells, with and without XAV-939 treatment, accompanied by representative images of tumor spheres. **(D)** *In vivo* bioluminescent imaging and quantification of xenograft tumor burdens were carried out in mice bearing ECs-GBM cells from the co-cultured group, with or without XAV-939 treatment. Data are means ± SD. ns: no significance; *, $P < 0.05$.

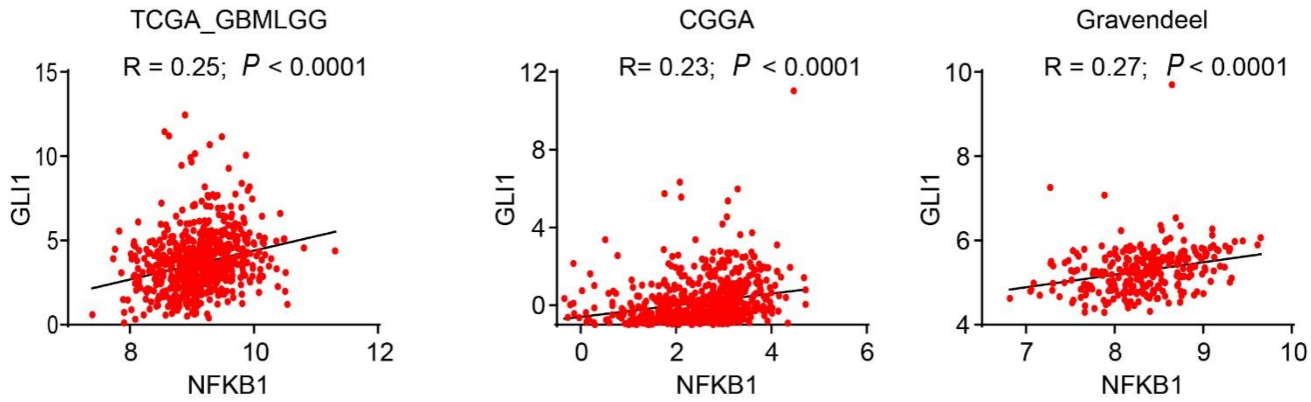

Figure S7 The correlation between expressions of NFKB1 and GLI1 in GBM databases.

The correlation plots showing relevance of NFKB1 to GLI1 from TCGA_GBMLGG, CGGA and Gravendeel datasets.

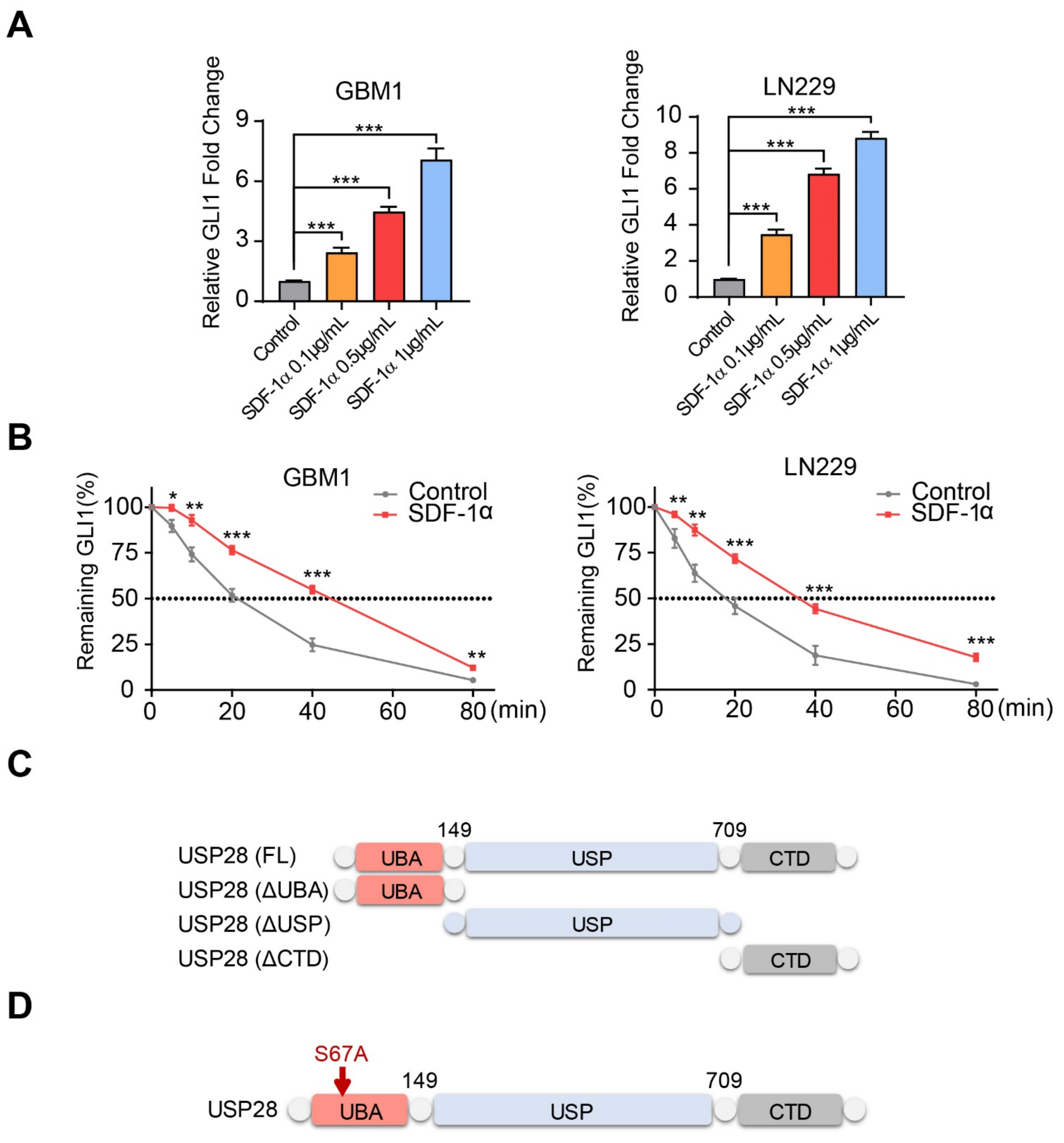

Figure S8 **SDF-1α activates USP28 to regulate the turnover of GLI1 protein.**

(A) qRT-PCR assay tested the expression of GLI1 in GBM cells treated with different concentrations of SDF-1α recombinant protein. **(B)** The halftime of GLI1 in GBM cells treated with SDF-1α recombinant protein or not at the indicated time. **(C)** Schematic representation of USP28 truncation mutants. **(D)** Schematic representation of USP28 point mutant

Table S1 The sequences of mRNA primers used in qPCR.

Gene	Forward primer (5' to 3')	Reverse primer (5' to 3')
β-actin	AGATGTGGATCAGCAAGCA	GCGCAAGTTAGGTTTTGTCA
SOX2	ACATGAACGGCTGGAGCAACG	CTGCGAGCTGGTCATGGAGTTG
CD133	TCGCCTCAAGCCAGCCTCAG	ACTTGGTGCCTCCTGCCTCAG
OCT4	CTGGGTTGATCCTCGGACCT	CCATCGGAGTTGCTCTCCA
NANOG	TTTGTGGGCCTGAAGAAAACT	AGGGCTGTCCTGAATAAGCAG
GLI1	AGCGTGAGCCTGAATCTGTG	CAGCATGTACTGGGCTTTGAA
